# Implications of sample size and acquired number of steps to investigate running biomechanics

**DOI:** 10.1038/s41598-021-82876-z

**Published:** 2021-02-04

**Authors:** Anderson Souza Oliveira, Cristina Ioana Pirscoveanu

**Affiliations:** Department of Materials and Production, Aalborg University, Fibigerstræde 16, Building 4, 9220 Aalborg E, Denmark

**Keywords:** Bone quality and biomechanics, Musculoskeletal system, Risk factors

## Abstract

Low reproducibility and non-optimal sample sizes are current concerns in scientific research, especially within human movement studies. Therefore, this study aimed to examine the implications of different sample sizes and number of steps on data variability and statistical outcomes from kinematic and kinetics running biomechanical variables. Forty-four participants ran overground using their preferred technique (normal) and minimizing the contact sound volume (silent). Running speed, peak vertical, braking forces, and vertical average loading rate were extracted from > 40 steps/runner. Data stability was computed using a sequential estimation technique. Statistical outcomes (*p* values and effect sizes) from the comparison normal *vs* silent running were extracted from 100,000 random samples, using various combinations of sample size (from 10 to 40 runners) and number of steps (from 5 to 40 steps). The results showed that only 35% of the study sample could reach average stability using up to 10 steps across all biomechanical variables. The loading rate was consistently significantly lower during silent running compared to normal running, with large effect sizes across all combinations. However, variables presenting small or medium effect sizes (running speed and peak braking force), required > 20 runners to reach significant differences. Therefore, varying sample sizes and number of steps are shown to influence the normal vs silent running statistical outcomes in a variable-dependent manner. Based on our results, we recommend that studies involving analysis of traditional running biomechanical variables use a minimum of 25 participants and 25 steps from each participant to provide appropriate data stability and statistical power.

## Introduction

There is a growing concern regarding the actual quality and relevance of published studies in all research fields. Factors such as low reproducibility and non-optimal sample sizes have been at the forefront of discussions in different research areas^1,2^. However, increasing concerns have been recently reported for human movement biomechanics, where sample sizes and the number of events recorded (trials or steps) might be insufficient to support the tested hypotheses^2–5^. On the core of this issue is the tradeoff between experiment duration and ecological validity, as the scientific community has no standardized experimental protocols to acquire “natural” human movement. Cyclical activities such as walking and running consist of repeating a defined movement pattern^1,6^. However, identical kinematics/kinetics performance in cyclical movements, like walking and running, are unlikely for humans due to intrinsic and extrinsic factors, such as equipment, surface, and/or metabolism^1,6^. Therefore, acquiring a number of repetitions (steps) to represent the variability of such a movement pattern is crucial to assure ecological validity. Calculating the peak of averaged data segments representing trials/steps is therefore an “acceptable” approximation of the recorded natural pattern^7^. However, calculating the average across the peaks identified from individual trials/steps provides better data representations^8,9^.

Studies regarding running biomechanics in the past 20 years have been defining the number of running steps based on previous literature. For instance, a guideline to achieve 90% statistical power in sports biomechanics proposed that by evaluating 5, 10, or 20 participants would require 10, 5, and 3 steps respectively^3^. This early recommendation has been extensively used in running biomechanics for various types of biomechanical variables^2,10–12^. Recommendations for an optimal number of recorded steps have been proposed using sequential estimation technique (SET)^2,7^, from which the ideal number of steps range from 13 to 20^2,11^. However, these variables may not represent the variability contained in running kinematics/kinetics. Inter-stride running variability is related to fluctuations in the control of motor function^13^ and the inherent complexity of the motor system^10,13^. As human locomotion can present considerable variability, it is highly relevant to take such factors into account when defining the amount of data to be acquired for running biomechanical studies.

In sports biomechanics, there are two types of parameters: global parameters, defined as the output of the human system as an entity (i.e., speed, contact time, step length, center of mass); and localized parameters, defined as output related to movement performance (i.e., joint angles, moments, power, ground reaction force)^1^. Furthermore, a decreased variability of a global parameter does not directly imply reduced variability in movement performance parameters^1^. Therefore, it has been suggested that special attention needs to be devoted to the variability of both global and localized parameters in studies related to sports biomechanics^1,6^. The coefficient of variation (CV) of global parameters such as running speed can vary between 2^14^ and 760%^15^, whereas foot contact time varies between 3.8^16^ and 10%^17^ (*see* Supplementary Table 1*for details on each variable*). However, localized parameters may present greater variability. The CV from the vertical loading rate ranges from 12^18^ to 109%^17^, whereas the CV from foot angle at initial contact ranges from 24^19^ up to 3288%^17^. These statistics suggest that different variables in running biomechanics present specific variability patterns. Therefore, defining the number of steps and/or sample size for a running experiment based on only one variable may affect the results of all other recorded variables.

In a short literature review, we included 56 manuscripts evaluating uninjured runners from the year 2000 to 2019 (Supplementary Table 2). In general, running biomechanics studies reported data with an average of 12 running steps per runner (median: 5; range: 3–200, Fig. 1A), and 51 out of 56 studies (91%) reported results with up to 10 steps. Only 5 out of 56 studies (9%) in our review used more than 10 steps to describe running performance. Interestingly, no clear arguments were found in the literature to justify the need for a large number of steps in running biomechanics. Moreover, studies comprising of multiple variables of different nature (kinetics, kinematics, neurophysiological) defined the number of trials or steps based on one of the variables investigated. Therefore, although establishing adequate sample sizes and number of steps analyzed is considered a priority in biomechanical studies^20^, the differences across variables might have been overlooked until present days.

**Figure 1:**
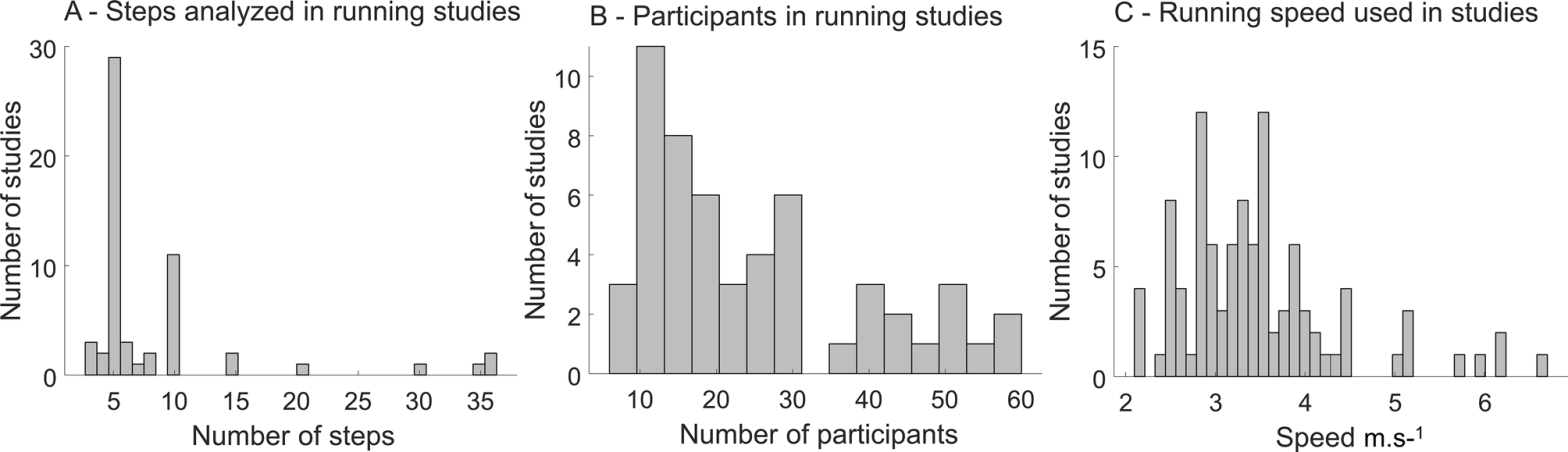
Distribution of running biomechanics studies describing the number of steps (**A**), participants (**B**), and running speed (**C**) from 2000 until present days. In total 60 studies were included in a short literature review (see Supplementary File 1). Some studies were not included in the plots to maximize visualization quality: In (**A**) Wouda et al. (2018)^43^ reported around 200 steps in their analysis. In (**B**) Bredeweg et al. (2013)^44^ and Stiffler-Joachim et al. (2019)^45^ reported 170 and 210 participants, respectively. In (**C**) Stiles et al. (2013)^46^ reported a running cadence of 130–140 steps/min, respectively 145–160 steps/min, as well as Morin et al. (2011)^47^, Rowlands and Stiles^48^(2012) and Pollard et al. (2018)^49^ used the preferred running speed of the participants.

Another relevant factor that assures an adequate statistical power of biomechanics research is establishing an adequate sample size^4^. Unfortunately, studies with a small sample might lead to a decreased statistical power, poor reliability, and inflated effect size^4,10^, whereas studies with large sample sizes may be more expensive and may subject their participants to unnecessary interventions^21^. The running biomechanics studies we reviewed reported results using 31 runners on average (median: 21; range 8–210 runners, Fig. 1B), with 8 out of 56 studies (14.3%) that reported data from only 10 runners. Previous review papers in biomechanics have shown a median sample size of 14.5^10^ or ranging from 12 to 18 participants^4^. It seems that researchers are increasing the number of runners included in their studies within the last two decades. However, more research is necessary to further establish normative guidelines regarding data acquisition factors such as sample size and number of steps for research in running biomechanics. Similarly, the running speed is widely variable in running biomechanics studies, from which the mean value is 3.46 m s^−1^ (median: 3.33 m s^−1^; range: 2.13–6.7 m s^−1^, Fig. 1C). Running speed can dramatically influence running kinematics and kinetics^22^, which requires caution when comparing results from studies with non-comparable running speed. Therefore, understanding the implications that may arise when selecting varying sample sizes, number of events (trials or steps), and locomotion speed is highly relevant in designing experimental biomechanics studies.

Therefore, the purpose of this study was to answer two main questions: (1) how to select an appropriate number of steps and sample size to conduct a study regarding running biomechanics involving multiple variables? (2) What are the implications of the selected number of steps and sample sizes on data variability and statistical outcomes? To answer these questions, a simple intervention consisting of reducing the sound volume produced from running footfalls was applied in this study. The silent running performance was compared to normal running at a similar running speed. This intervention was implemented to induce expected changes in running kinematics/kinetics . For instance, it is known that silent runnning significantly reduces loading rate and peak vertical forces^23,24^, however, no changes are found for running active peak and foot contact time^24^. A sample of 44 runners performing 40 steps in each condition was used to investigate the implications of using several combinations of reduced sample sizes and steps on data variability [sequential estimation technique (SET) and CV], as well as statistical outcomes (*p* values and effect size from the comparison between normal vs silent running).

## Methods

### Participants

Forty-four healthy recreational runners (8 females and 36 males, age: 26.81 ± 4.04 years, weight: 77.29 ± 11.76 kg, height 178.41 ± 9.34 cm, BMI 24.20 ± 2.74) agreed to participate in our study. Runners reported a moderate to a high level of physical activity according to the International Physical Activity Questionnaire (3269.36 ± 1542.92 MET) or with a minimum level of running experience of 3 years and weekly mileage of 13.9 ± 9.1 km. None of the participants had any history of lower extremity musculoskeletal injury or running-related injuries during the testing or in the previous six months. All the participants provided verbal and written informed consent before participating. All experimental methods were carried out in accordance with relevant guidelines and regulations, and the experimental procedures of the present study were in accordance and approved by the Ethical Committee of North Jutland (Region Nordjylland).

### Experimental procedure

The experiment consisted of a single session where participants performed two different running tasks: normal running, as well as running while minimizing the sound volume of their footfalls (silent running). The running tasks were performed overground, allowing for the recording of ground reaction forces and motion capture. Familiarization procedures were conducted prior to data recordings, as well as during the session for the silent running condition. The order for performing normal and silent running was randomized across all participants.

### Running tasks

Participants performed a 5-min warm-up consisting of walking lunges, skipping, leg swings, and run-throughs (Phan et al. 2017). Following the warm-up, participants spent 5–10 min familiarizing themselves with the running track and established their preferred running speed. A 20-m indoor overground obround running track (2-m running turns and 8-m sprinting lines) was used. A force platform and four microphones surrounding it were located in the middle of one sprint line. The microphones did not interfere with the runner’s sprint-line trajectory. All runners were provided with a standard running shoe (Nike Air Pegasus) to perform the familiarization and testing procedures.

For the normal running, participants were instructed to run as naturally as possible at a self-selected speed, aiming to hit the force plate with the right foot. For the silent running, participants were instructed to actively minimize the volume of sound produced during their running footfalls throughout the track, especially at the sprint line containing the force platform and microphones. Runners were not instructed regarding how they should change their running technique to achieve silent running. Participants performed both normal and silent running conditions in three sets of 3-min for each condition. Participants were instructed to run continuously around the track at their preferred running speed while stepping with the right foot on the force plate at every lap. As participants ran at their preferred speed, the number of laps in the track varied across participants (between 14 and 19 laps per set). In order to attenuate the effects of fatigue in the experiment, a 3-min rest period was offered to runners between each recording. The running speed was monitored on a trial-by-trial basis by a researcher recording the time (and subsequently the speed) runners took to pass through a 3-m sector of the track that contained the force plate in the middle. This speed monitoring allowed instructing runners regarding changes in their running speed throughout a set, as well as between sets and between conditions. In case the running speed changed 10% above or below the pre-established value, runners were instructed to increase or reduce their pace in the next lap.

### Data collection

Data were recorded on an indoor motion capture laboratory, on a 20-m obround running track. The track had an 8-m sprinting line with a mid-data collection landmark consisting of a floor embedded force plate (AMTI Optima Gen 5, Watertown, MA, USA), sampled at 1000 Hz that provided three-dimensional forces and moments. Moreover, an 8-camera motion capture system (Qualisys 1.0, Qualisys Oqus, Sweden) sampled at 200 Hz was used to record position data from 8 retro-reflexive markers (16 mm diameter). Four of these markers were fitted equidistantly onto an elastic strap that was positioned on the participant’s head, and the remaining four markers were positioned on the right shoe at the foot tip, heel, medial, and lateral metatarsal heads of the running shoe following previous literature^25^. Both running kinetic and kinematic data were synchronously captured through the motion capture software.

### Data analysis—variables extraction

Customized scripts on Matlab (2019b, The Mathworks, Natick, USA) were used to analyze kinetics and kinematics data. Individual running steps were defined using the raw vertical ground reaction force data, defining foot contact to the force plate when the vertical force exceeded 25 N. Subsequently, the force data were filtered using a fourth-order Butterworth low pass filter (60 Hz cut-off frequency) and normalized to the participant’s body weight (%BW). Foot contact time (FCT), peak vertical force (PKF), anterior–posterior peak braking force (BRK), and vertical loading rate, which was defined as the slope of the line between 20 and 80% of the impact peak^26–28^, were extracted from the force data. If the impact peak was not found, a pre-set point in time, 13% of the total contact time was used instead^26,27,29–31^.

The participants running speed was calculated within a range of one meter before and after stepping onto the force plate, by computing the average displacement across all head markers for each successful running cycle. The foot contact angle (FCA) was defined as the angle between the vector from the forefoot marker to the heel marker with respect to a vector representing the anterior–posterior direction in the laboratory coordinate system. The FCA was offset by the angle between these two vectors at 30% of the stance phase when the foot made full contact with the force plate. Positive FCA values were achieved when the heel marker was higher than the toe marker and negative values when the toe marker was higher.

### Data analysis—sequential estimation technique (SET)

SET determines the point of mean stability of a variable recorded continuously and was implemented in the present study following previous literature^11^. Then, the sequential data points from all 40 steps were averaged to create the target stability, from which a bandwidth of ± 25% was used to determine whether the individual steps fell within this bandwidth. Subsequently, a moving point mean was calculated, starting from the first two points and increasing after each interaction by one point until the total amount of steps was reached. The point of stability is the first point that is within the bandwidth along with all subsequent points recorded. SET was applied in all variables to define their point of stability. Therefore, data average stability was defined as the average stability of the individual participant for each parameter investigated where the number of steps analyzed should not influence the statistical outcome.

### Data permutation (number of steps vs number of runners)

Although each participant performed a different number of valid steps on the force plate, they all successfully performed at least 40 valid steps. The data from these 40 steps across all 44 participants was defined as the full running dataset, which would then be compared to sub-samples of steps (sequences of 5, 10, 15, 20, 25, 30, 35, and 40 steps), combined with sub-samples of participants (random samples of 10, 15, 20, 25, 30, 35 and 40 runners). As an example, the loading rate was computed for 10 runners performing 5 steps. There are ~ 2.5·10^10^ possible combinations of 10 runners from a total of 44 runners. Moreover, there are 658,008 possible combinations of 5 steps from a total of 40 steps for each runner. Therefore, for our example there are ~ 1.6·10^16^ possible combinations to represent 10 runners performing 5 steps each. From this total, 100,000 combinations were randomly selected from both normal and silent running (with the same runners in both conditions). Moreover, the number of steps was averaged for each runner and subsequent statistical comparison between normal and silent running was conducted for each combination. The colormaps in the results section show the average across the 100,000 statistical comparisons. Forrester et al. (2015)^2^ used 10,000 different samples to run simulations and reach relevant conclusions. Preliminary calculations demonstrated that the use of 100,000 permutations was enough to represent similar variability as ≥ 10 million number of permutations. Therefore, data from the six dependent variables (foot contact time, loading rate, peak vertical force, peak braking force, running speed, and foot contact angle) were permuted for both normal and silent running.

### Statistical analysis

Differences between normal and silent running using the standard dataset (44 runners, 40 steps per runner) for all dependent variables were assessed using two-tailed paired t-Student tests. Cohen’s D effect size was computed for all comparisons. Similarly, the 100,000 paired samples were compared using similar t-Student tests, and the respective Cohen’ D effect size (“small” values around 0.2, “medium” for 0.5, and “large” above 0.8^26^) were computed for each pair. In addition, interquartile ranges (IQR, the difference between the 25th and 75th percentile from a given sample) and coefficient of variation (CV) were computed from all 100,000 samples of both conditions. Furthermore, a sample size estimation was conducted following the formula:

1 $$n=\left[\frac{{z}^{2}*p*(1-p)}{{e}^{2}}\right]/[1+\frac{{z}^{2}*p*(1-p)}{{e}^{2}*N}]$$

where *z* = 1.96 for a confidence level of 95%, *p* = 0.7 proportion of the population, *N* = 44 population size and *e* = 0.05 the margin of error^32^. A recommended sample size of 39 participants was found.

## Results

### Full running dataset analysis (44 runners, 40 steps/runner)

**Figure 2:**
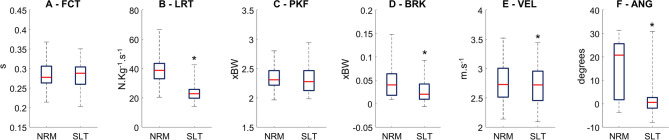
Comparison normal (NRM) vs silent running (SLT) for foot contact time (FCT, **A**), vertical loading rate (LRT, **B**), peak vertical force (PKF, **C**), peak braking force (BRK, **D**), running speed (VEL, **E**) and foot contact angle (ANG, **F**). Box plots represent 25th and 25th percentiles, and data range (*dashed vertical lines*) during normal (NRM) and silent running (SLT). * denotes a significant difference in relation to normal running (*p* < 0.05).

There were significant reductions in loading rate (*p* < 0.0001, effect size: 1.41, Fig. 2), peak braking force (*p* < 0.0001, effect size: 0.66), running speed (*p* < 0.05, effect size: 0.167) and foot contact angle (*p* < 0.0001, effect size: 1.03) during silent running when compared to normal running. No changes were found for foot contact time and peak vertical force (*p* > 0.05).

### The interquartile range across different number of steps

**Figure 3:**
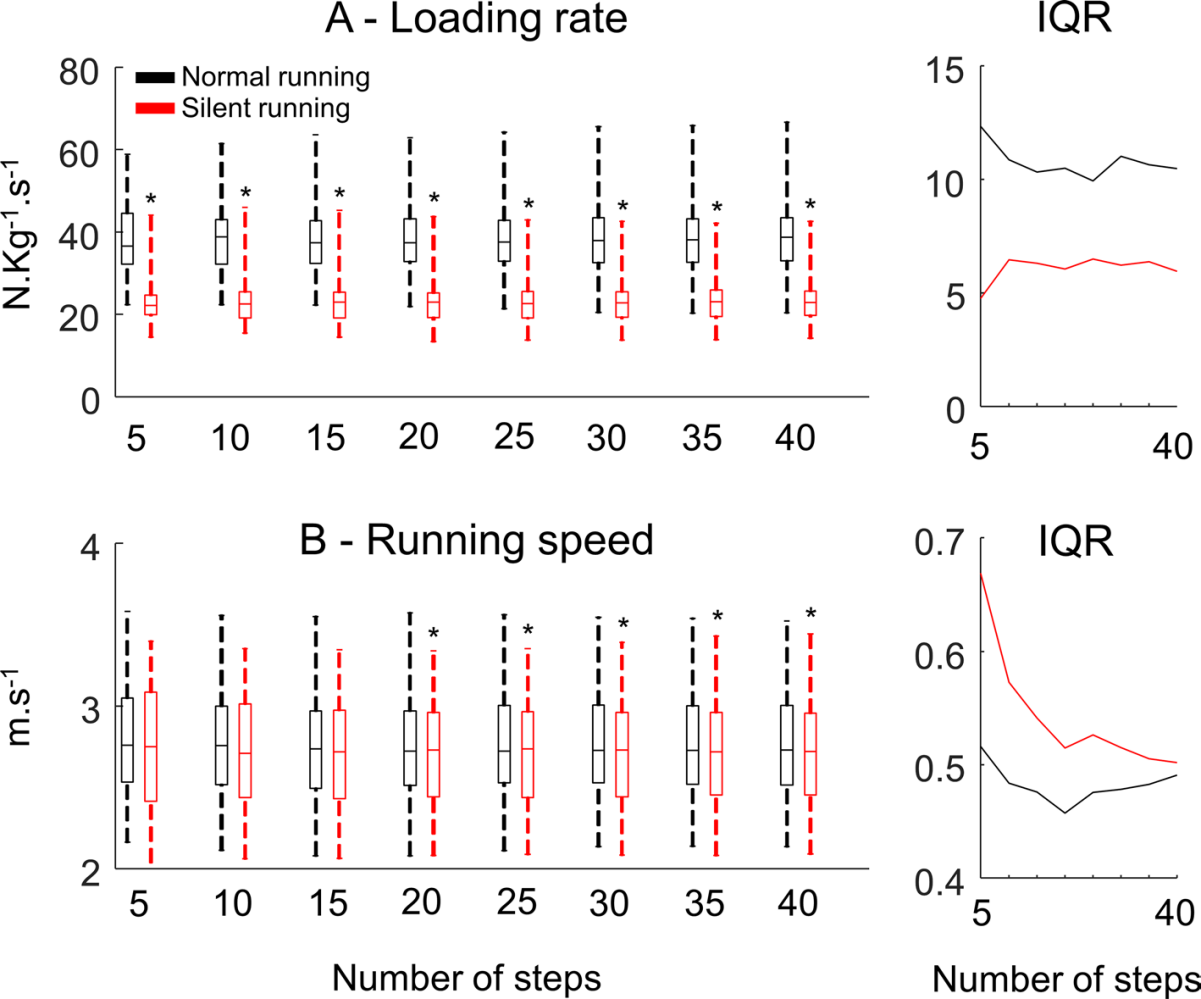
Comparison normal (*black*) versus silent running (*blue*) for vertical loading rate (**A**), and running speed (**B**). Box plots represent 25th and 25th percentiles and data range (*dashed vertical lines*) across different numbers of steps. On the right, interquartile ranges (IQR) across the different number of steps. * denotes a significant difference in relation to normal running (*p* < 0.05).

An illustration of such distribution using the loading rate (Fig. 3A) and running speed (Fig. 3B) depicts no substantial changes in data distribution when comparing data from 5 to 40 steps using all 44 runners. This pattern was maintained across all variables. Interestingly, the IQR may show differences both across the number of steps as well as between conditions. In particular, running speed presented a reduction in IQR that resulted in significant differences when using 20 steps or more. All other variables presented consistent IQR across the number of steps.

### Sequential estimation technique

**Figure 4:**
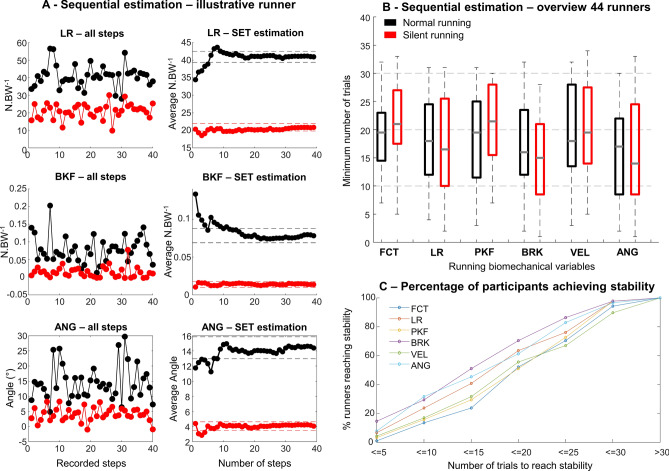
Illustration of sequential estimation to determine the number of steps needed to achieve stability from 10 (left) and 40 runners (right) during both normal (black) and silent running (red). (**A**) Within parameter variability of a representative runner (left) and sequential estimations (right) for loading rate (LR), peak braking force (BKF), and foot contact angle (ANG). (**B**) Box plots represent 25th and 75th percentiles, and data range (*dashed vertical lines*) for multiple parameters (foot contact time—FCT, loading rate—LR, peak vertical force—PKF, peak braking force—BRK, running speed—VEL, foot contact angle—ANG). (**C**) The linear plots illustrate parameter variability and the percentage of participants as well as the number of steps required to achieve stability across both normal and silent running.

SET was used to determine the minimum number of trials required to reach a stable data average within 40 steps. It was evident that different variables may present different distribution over time (Fig. 4A), therefore influencing the minimum number of steps required to reach stability. Furthermore, Fig. 4A—right panels, illustrate the within parameter variability of one illustrative runner, where sequential estimation shows how increasing the total number of steps analyzed decreased the within step variability and increases parameter stability. An evaluation across all 44 runners demonstrated that the average number of steps to reach stability ranges from 15 ± 8 (peak braking force) to 20 ± 7 (foot contact time). Moreover, the inter-subject variability is high across all variables and conditions (Fig. 4B), in which the 75th percentile is above 20 trials across all variables. The percentage of runners requiring 10 or fewer steps to reach stability is below 30% of the overall sample of runners (Fig. 4C), and this number is variable dependent on the variable. Most of the running variables require 15 to 20 steps to reach stability. It was determined that at least 20 steps are needed to reach more than 50% of the sample of runners with a stable average.

### Data distribution—10 vs 40 runners

**Figure 5:**
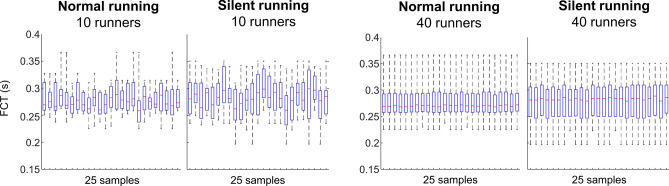
Illustration of 25 subsamples containing foot contact time (FCT) from 10 (left) and 40 runners (right) during both normal and silent running. Box plots represent 25th and 75th percentiles, and data range (*dashed vertical lines*). The 25 subsamples were randomly selected from 100,000 paired samples generated for further analysis.

Figure 5 demonstrates that using 10 runners can drastically influence the representation of the data for both conditions, compared to 40 runners. Box plots, median values, and range across random sub-samples vary substantially when only using 10 runners, and this effect is substantially reduced using 40 runners.

### Effect of number of runners and steps on normal vs silent comparison

#### Foot contact time

**Figure 6:**
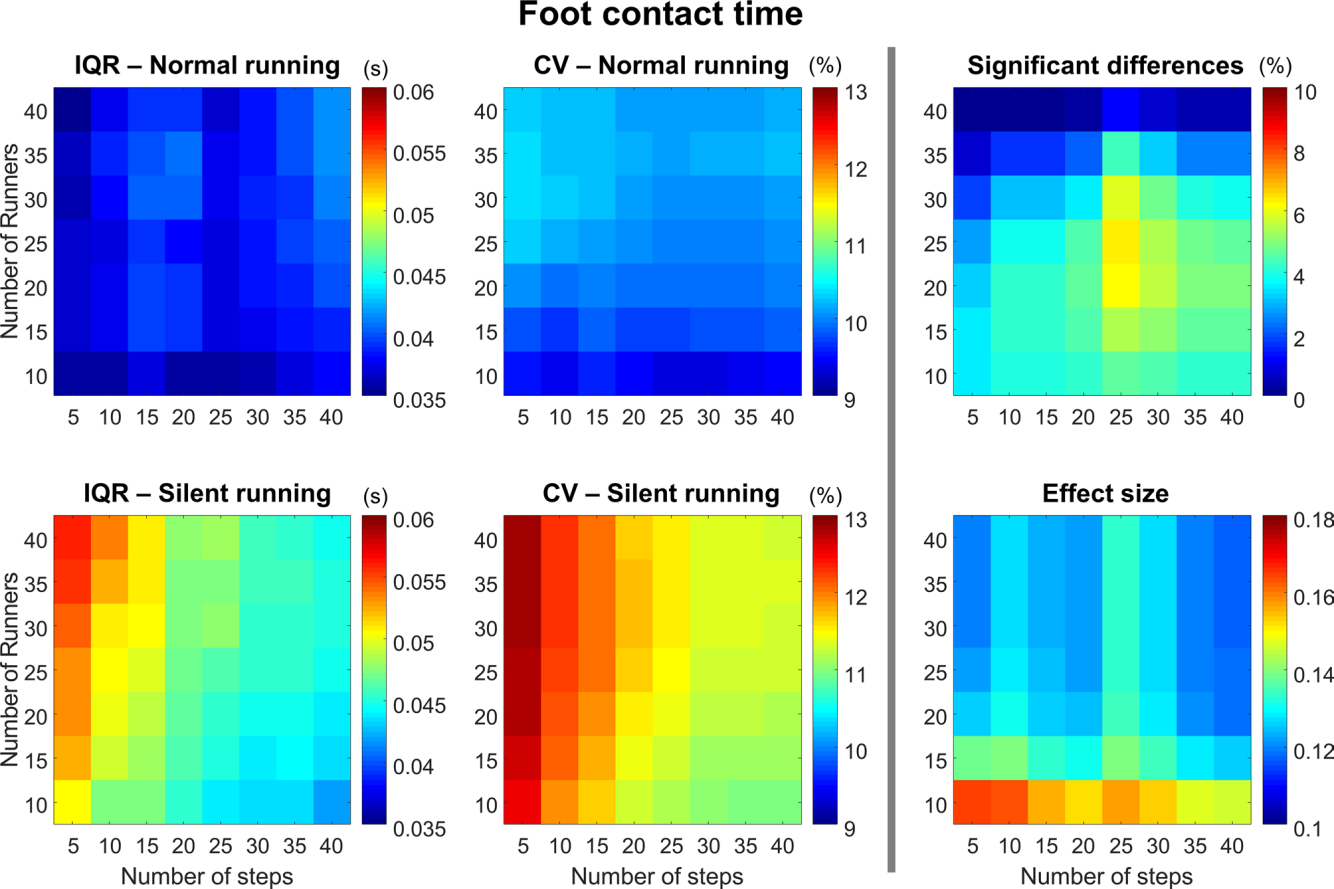
Colormaps demonstrating foot contact time interquartile range (IQR, *left column*) and coefficient of variation (CV, *central column*) from the combination of 10–40 runners versus 5–40 running steps. Data is shown from normal running (*top panels*) and silent running (*bottom panels*). Each value is an average from 100,000 paired permutations for each combination. The number of significant differences (*p* < 0.05, expressed as a percentage of 100,000 comparisons, *top right panel*) and their respective effect size (*bottom right panel*) illustrate the changes in statistical outcomes depending on the combination of number or runners versus number of steps.

The foot contact time presented greater IQR (Fig. 6, *left column*) and CV (Fig. 6, *central column*) for the silent condition when compared to normal. Overall, the CV is low (< 15%); however, for the silent condition, the CV is reduced when a greater number of steps are used. The number of runners does not influence the CV. However, the lower the number of runners, the higher the probability of finding significant differences (Fig. 6, *top right panel*). The effect size (Fig. 6, *bottom right panel*) is generally low across all combinations, but it tends to be greater when using a lower number of participants (below or equal to 15).

#### Peak vertical force

**Figure 7:**
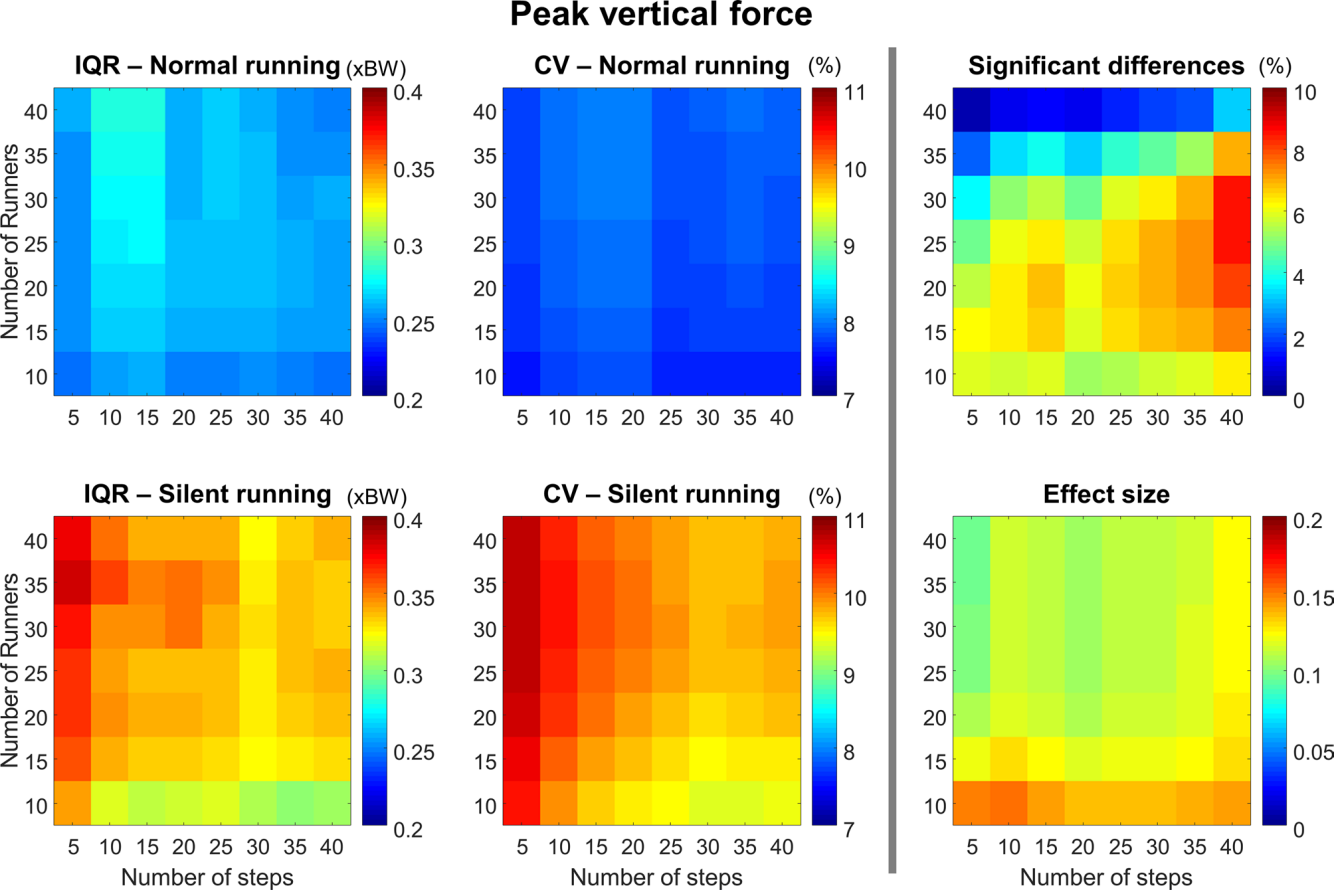
Colormaps demonstrating peak vertical force interquartile range (IQR, *left column*) and coefficient of variation (CV, *central column*) from the combination of 10–40 runners versus 5–40 running steps. Data is shown from normal running (*top panels*) and silent running (*bottom panels*). Each value is an average from 100,000 paired permutations for each combination. The number of significant differences (*p* < 0.05, expressed as a percentage of 100,000 comparisons, *top right panel*) and their respective effect size (*bottom right panel*) illustrate the changes in statistical outcomes depending on the combination of number or runners versus number of steps.

The peak vertical force resulted in greater IQR and CV for the silent condition when compared to normal (Fig. 7, *left and central columns*). Overall, the CV is low (< 15%); however, for the silent condition the CV is reduced with fewer runners (< 20) and with a greater number of steps. There was a greater probability to find significant differences when using 15–30 runners with more than 30 steps. However, the effect size was usually small (< 0.15).

#### Peak braking force

**Figure 8:**
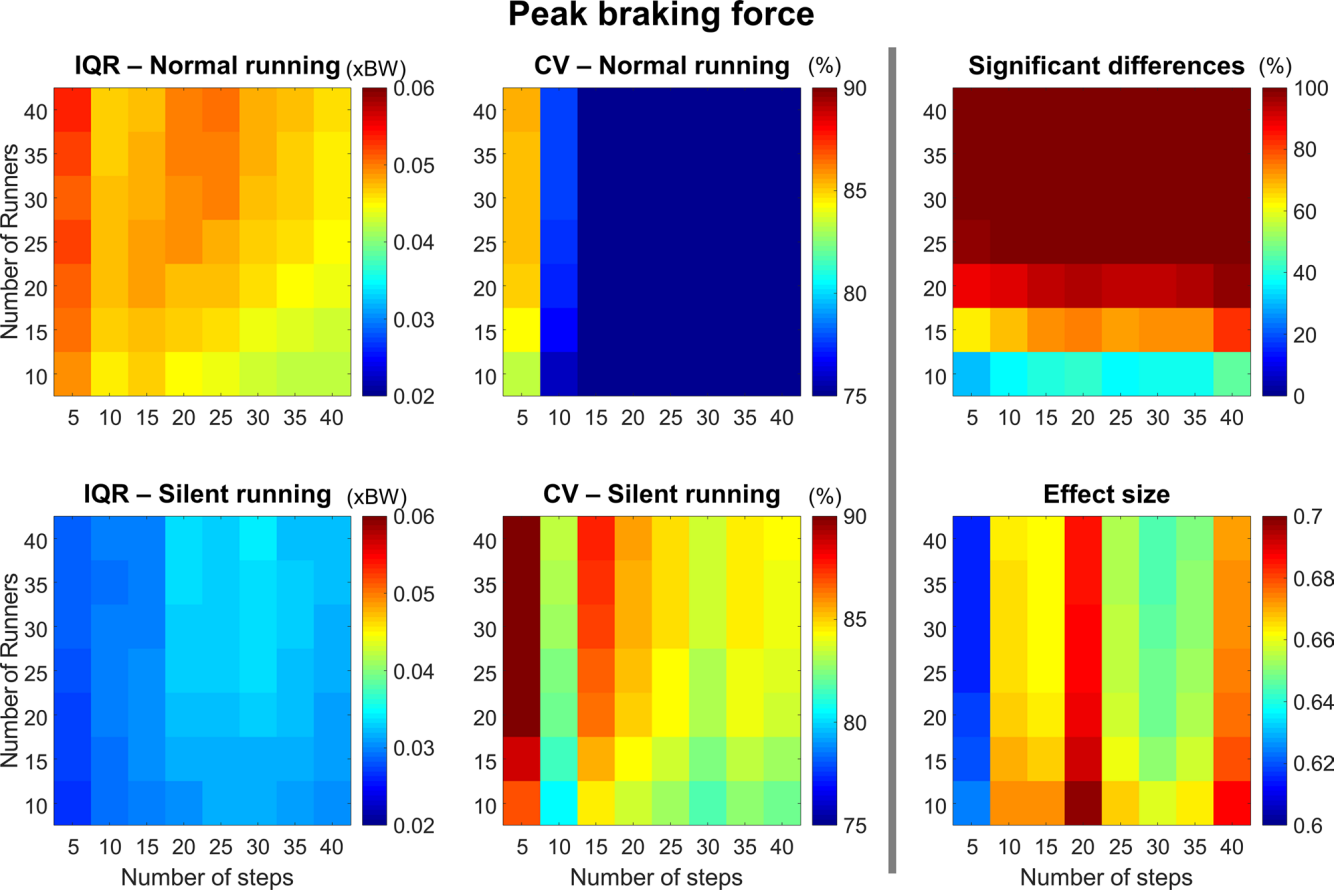
Colormaps demonstrating peak braking force interquartile range (IQR, *left column*) and coefficient of variation (CV, *central column*) from the combination of 10–40 runners versus 5–40 running steps. Data is shown from normal running (*top panels*) and silent running (*bottom panels*). Each value is an average from 100,000 paired permutations for each combination. The number of significant differences (*p* < 0.05, expressed as a percentage of 100,000 comparisons, *top right panel*) and their respective effect size (*bottom right panel*) illustrate the changes in statistical outcomes depending on the combination of number or runners versus number of steps.

The peak braking force presented a greater IQR for the normal condition, which generally increased with a lower number of steps (Fig. 8, *left column*). Overall, the CV is high (> 60%), increasing with a lower number of steps (< 10 steps) when compared to the other step categories. No influence of the number of participants is seen. There was a greater probability to find significant differences when using > 20 runners, with no relevant effect of the number of steps on statistical outcomes. The effect size was moderate across all combinations (from 0.6 to 0.7).

#### Vertical loading rate

**Figure 9:**
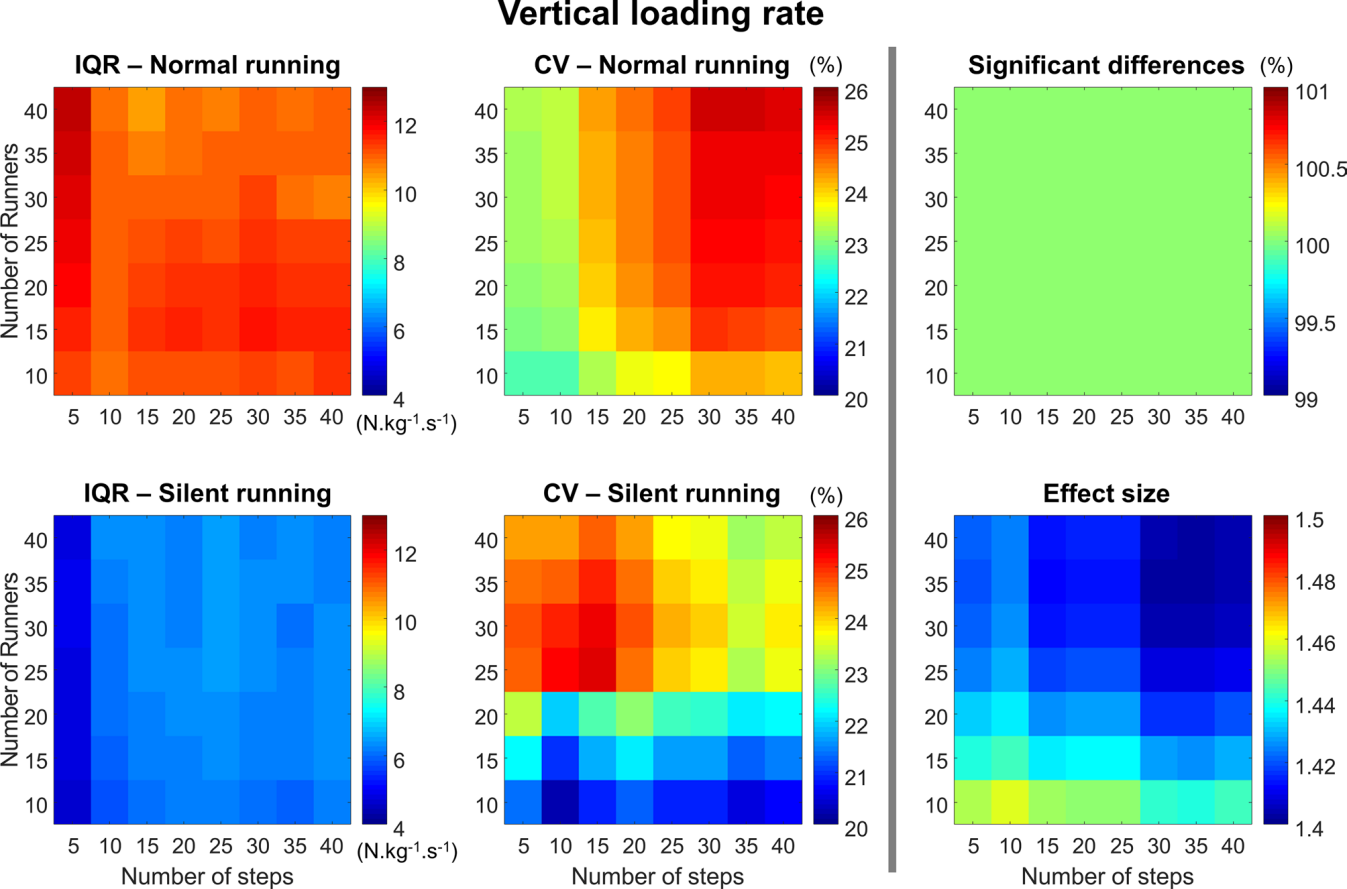
Colormaps demonstrating vertical loading rate interquartile range (IQR, *left column*) and coefficient of variation (CV, *central column*) from the combination of 10–40 runners versus 5–40 running steps. Data is shown from normal running (*top panels*) and silent running (*bottom panels*). Each value is an average from 100,000 paired permutations for each combination. The number of significant differences (*p* < 0.05, expressed as a percentage of 100,000 comparisons, *top right panel*) and their respective effect size (*bottom right panel*) illustrate the changes in statistical outcomes depending on the combination of number or runners *vs* number of steps.

The loading rate presented greater IQR for the normal running, with no influence of steps or sample size (Fig. 9, *left column*). The CV was influenced differently depending on the condition. The normal condition presented a greater CV when analyzing > 15 steps regardless of the number of runners used, whereas during silent running the CV increased when using less than 25 steps and more than 20 participants steps (Fig. 9, *central column*). Significant differences between conditions were found for any combination of steps and runners with an increased effect size (> 1.4). However, the effect size was lower when using > 30 steps and > 30 runners (Fig. 9, *right column*).

#### Running speed

**Figure 10:**
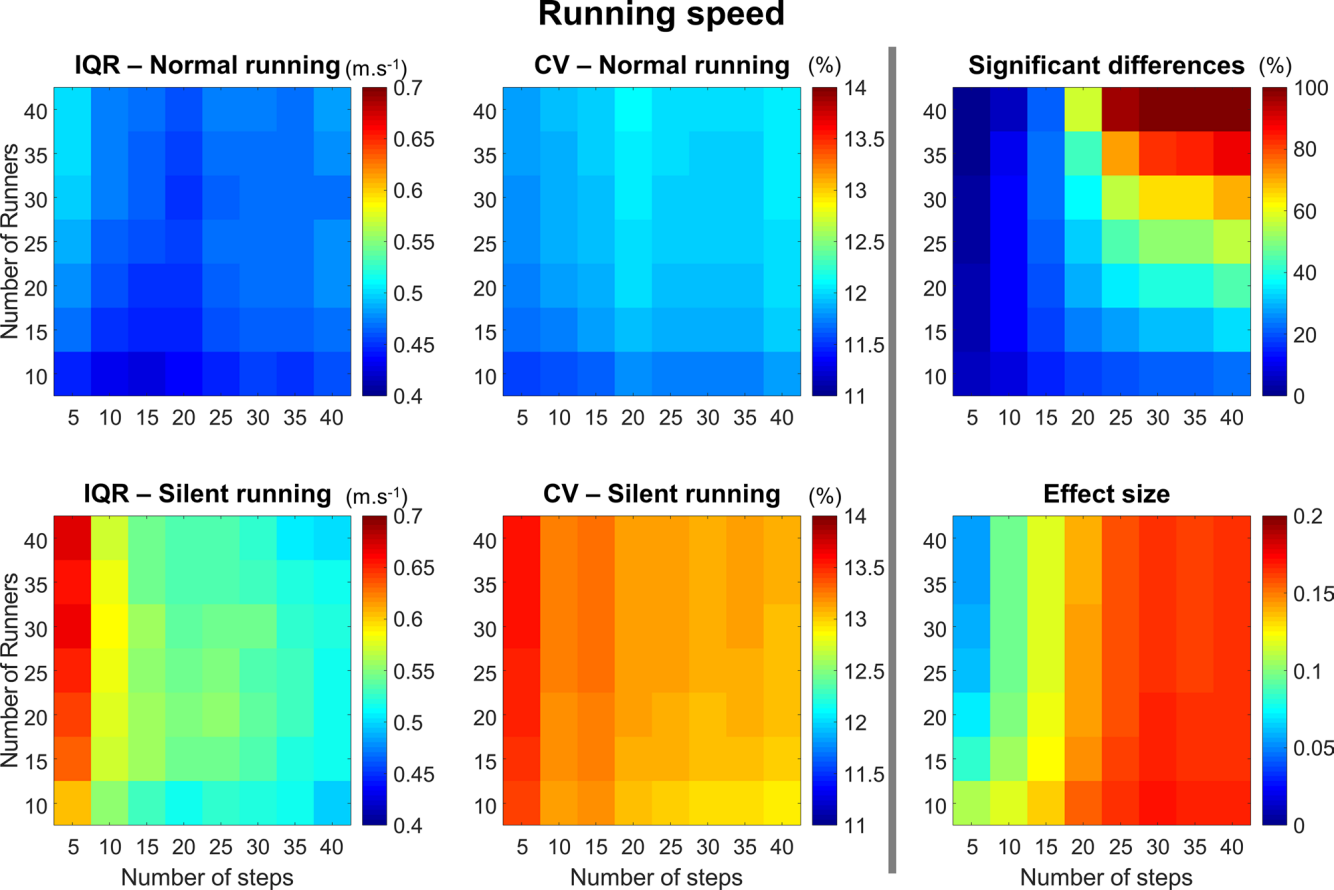
Colormaps demonstrating running speed interquartile range (IQR, *left column*) and coefficient of variation (CV, *central column*) from the combination of 10–40 runners versus 5–40 running steps. Data is shown from normal running (*top panels*) and silent running (*bottom panels*). Each value is an average from 100,000 paired permutations for each combination. The number of significant differences (*p* < 0.05, expressed as a percentage of 100,000 comparisons, *top right panel*) and their respective effect size (*bottom right panel*) illustrate the changes in statistical outcomes depending on the combination of number or runners versus number of steps.

The running speed presented a greater IQR for the silent running, resulting in greater IQR with fewer number of steps (Fig. 10, *left column*). The CV was generally low for both running conditions (Fig. 10, *left column*), with a tendency to increase when using a lower number of steps during silent running. The probability of finding significance was greater when analyzing > 30 runners with > 25 steps. However, using less than 15 steps dramatically reduced the probability of finding differences, regardless of the number of steps the runner used (Fig. 10, *right column*). Despite these differences, the effect size was, in general, small (< 0.2), increasing with the number of steps.

#### Foot contact angle

**Figure 11:**
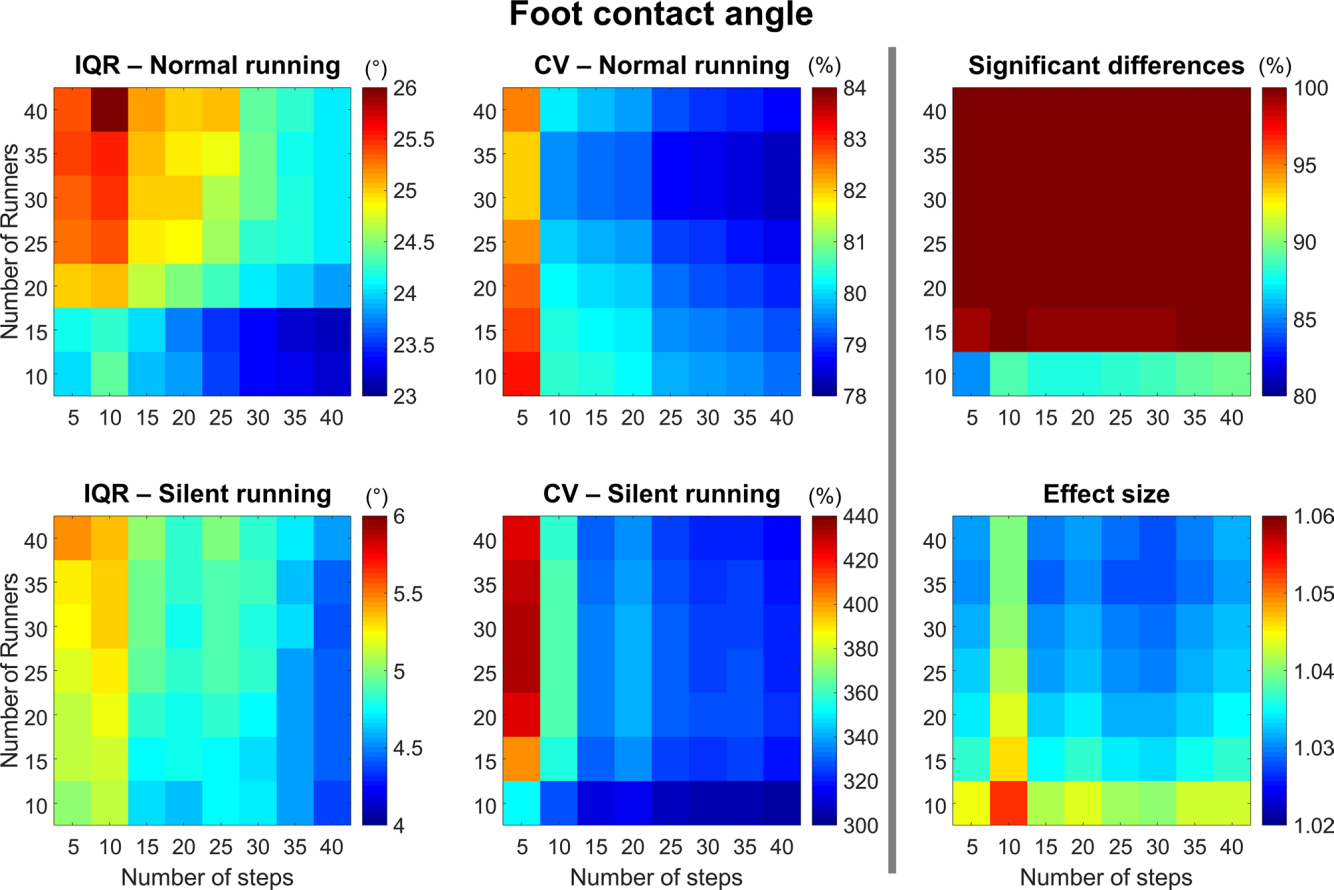
Colormaps demonstrating foot contact angle interquartile range (IQR, *left column*) and coefficient of variation (CV, *central column*) from the combination of 10–40 runners versus 5–40 running steps. Data is shown from normal running (*top panels*) and silent running (*bottom panels*). Each value is an average from 100,000 paired permutations for each combination. The number of significant differences (*p* < 0.05, expressed as a percentage of 100,000 comparisons, *top right panel*) and their respective effect size (*bottom right panel*) illustrate the changes in statistical outcomes depending on the combination of number or runners versus number of steps.

The foot contact angle presented greater IQR in the normal condition when compared to silent (Fig. 11, *left column*). Moreover, the IQR was greater when analyzing > 20 participants with 15 steps or less. The CV in both normal and silent conditions is greater when analyzing only 5 steps, with a low influence of the number of participants. The substantially greater CV during silent running is also worth noting. The probability of finding significant differences is assured by using more than 10 runners, regardless of the number of steps. The effect size is generally high (> 1.0) but tends to be lower when using more than 20 runners and 25 steps.

### Specific recommendations regarding sample size and number of steps

**Figure 12:**
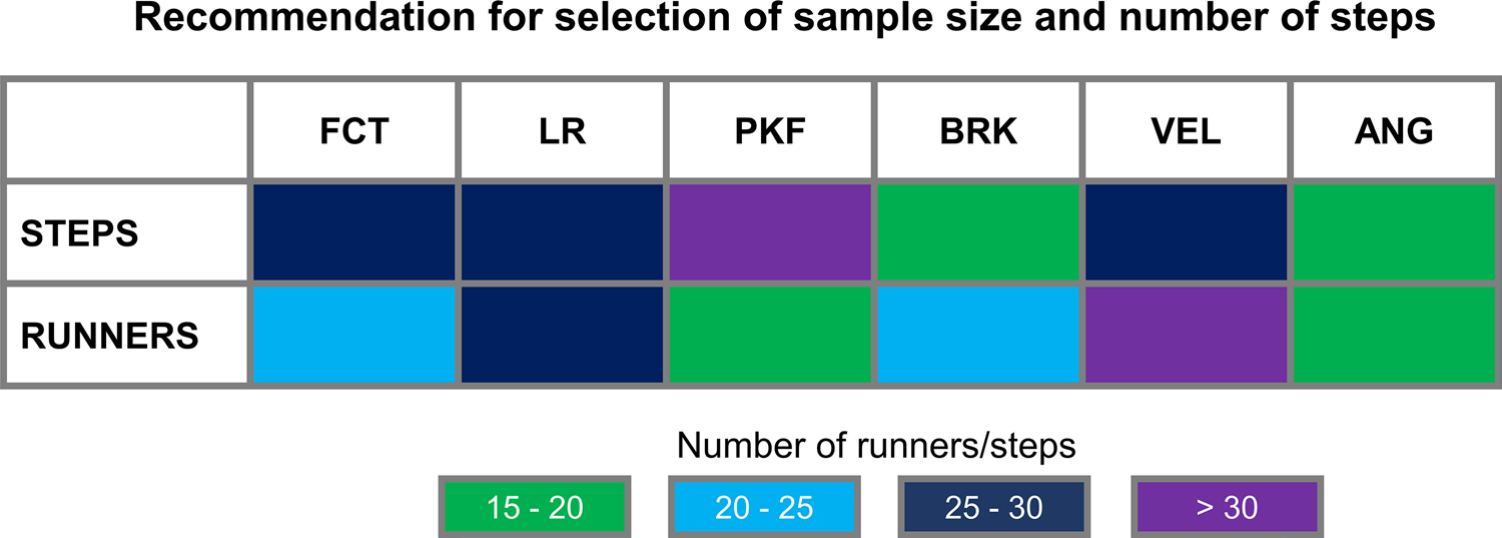
Colormap representing the recommended number of steps and sample size required to achieve individual stable average, as well as reduce variability and maximize effect size. *FCT* foot contact time, *LR* loading rate, *PKF* peak vertical force, *BRK* peak braking force, *VEL* running speed, *ANG* foot contact angle.

In Fig. 12, we summarized specific recommendations to the number of runners and number of steps to be recorded with respect to different running biomechanical variables. These recommendations were based on the results provided by the SET analysis and the permutation analysis across each variable.

## Discussion

The purpose of this study was to provide information on (1) how to select sufficient steps and sample sizes and (2) the implication of the selected sample sizes and steps on the variability and statistical outcomes of various running biomechanical variables. A total of 44 runners performing 40 steps in two running conditions (normal and silent) were used to explore the implications of using several combinations of sample sizes and steps on the variability of running biomechanical parameters. The main findings of our study were that: (1) stable averages across the investigated biomechanical variables require more than 10 steps for the majority of runners, as less than 35% of runners reached a stable average within 10 steps or less. (2) Variables expected not to be influenced by the silent running intervention, such as foot contact time and peak vertical force, presented increased likelihood of reporting significant differences when the sample size was ≤ 25 runners. (3) Running kinetic variables (vertical loading rate and peak braking force), which were expected to demonstrate significant differences, presented greater IQR for normal running. Nonetheless, vertical loading rate consistently presented significance and large effect sizes, whereas peak braking force reached an expressive probability of significance only when using more than 20 runners. Finally, (4) foot contact angle demonstrated substantial variability in the silent condition, especially when using less than 10 steps. Moreover, the foot contact angle presented an optimal probability of significance when more than 10 runners were analyzed. These results highlight the importance of defining the adequate number of steps and sample size with respect to a particular running biomechanics study, due to the fact that different variables may require different amounts of steps and/or runners. Therefore, it is recommended that studies involving analysis of traditional running biomechanical variables use a minimum of 25 participants to increase the likelihood of appropriate statistical power and large effect sizes. Moreover, acquiring data from at least 25 steps from each participant may increase the likelihood of achieving data average stability across different variables for the majority of the study sample. Our results may serve as guidelines to optimize experimental protocols in running biomechanics, as well as standardize minimum requirements for appropriate comparison between results achieved from different research groups worldwide.

An important outcome from our short literature review (see Supplementary Table 2) was that 91% of biomechanical studies (51 out of 56) draw scientific conclusions based on 10 or fewer steps. According to our findings, the stability of the mean is achieved for only 10–30% of runners when using up to 10 steps across the investigated biomechanical variables (see Fig. 4C). Moreover, the number of steps required to reach stability is dependent on the type of variable targeted. Some previous recommendations regarding the number of steps are based on simulations and not experimental data^2,3^. Our results suggest that a lower number of steps (< 10) and low sample size (< 20) may prevent finding significant differences for most of the variables. These results corroborate studies from Bates et al. (1992)^3^ and Forrester et al. (2015)^2^ that recommended evaluating 20 participants^3^ and approximately 20 steps^2^. However, Bates et al. (1992)^3^ compiled the recommendations using 1000 possible simulations based only on vertical ground reaction force data, disregarding differences across biomechanical variables. Furthermore, Forrester et al. (2015)^2^ generated recommendations solely based on breast displacement during running, which is a highly specific variable that might not fully represent the variability from classic running biomechanical variables. Salo et al. (1997)^33^ found that kinematic variables (e.g. hip, knee, and ankle angle of the lead foot) from sprint hurdle can achieve 90% statistical power using on average 6 steps for female sprinters (median: 3, range 1–61 steps) and 16 steps for male sprinters (median: 7, range 1–78). On the other hand, the same study^33^ demonstrated that the time of maximum angular velocity requires 61 steps to achieve 90% statistical power for females, and horizontal velocity lost requires 78 steps to achieve 90% statistical power for males. Thus, it has been a general consensus that movement variability is variable-dependent^2,33^, and the present study provides estimations of number of steps and sample size to reach data average stability and relevant effect sizes in different biomechanical variables. Moreover, our results further support the statement that variability is variable-dependent, but also that the outcomes of objective comparison (such as normal vs silent running) may be influenced by both sample size and number of steps. Therefore, our results represent an important advance towards standardizing experimental procedures to investigate different aspects of running biomechanics.

A positive aspect of movement variability is the possibility to achieve success on a motor action through several solutions^1^. However, either high or low variability may reveal the presence of a pathology, pain, overuse injuries, as well as demonstrate learning effects or environmental adaptations^1,6^. Early^3^ and more recent^2^ methodological research on this topic has shown that a reduced number of steps can increase the mean difference between conditions due to a greater variability while reducing the effect size and power^2^. Our results corroborate such a statement. For normal running, the IQR of running speed remains constant as a function of the number of steps while the IQR was inversely related to the number of steps during silent running (n = 44, Fig. 3B, right panel). Statistical differences were only achieved when analyzing at least 20 steps. However, 96.5% (54/56) of the studies from our review used less than 20 steps to describe running speed. Although the difference seems marginal between conditions, by providing enough data to minimize variability, it is possible to reach significance. Therefore, studies comparing conditions may have different outcomes depending on the number of steps used for such statistical analysis.

The silent running condition proposed in this study aimed to change a runner’s approach to performing the stance phase, eliciting major changes in movement kinematics to minimize the sound output from each foot strike. The IQR demonstrated distinct differences between normal and silent running depending on the variable investigated in this study. Moreover, the manipulation of sample size and number of steps can influence IQR considerably. Variables such as foot contact time and peak vertical force presented greater IQR for silent running, mostly due to the different motor strategies participants used to perform the task. Interestingly, significant differences in running speed between normal and silent running were found when using more than 20 steps and 20 runners. Runners were expected to maintain their running speed across both conditions. In fact, the difference when using all 44 runners was on average 0.04 m.s^−1^ (~ 3% difference across all different numbers of steps). Moreover, the effect size across all runners versus steps combinations was low (maximum 0.2), suggesting that the differences might not be relevant despite being statistically significant. Our data revealed that the IQR for silent running speed gets lower as we increase the number of steps when using all 44 runners (see Fig. 3B), which may be the driver of statistical differences for such a large sample size. When evaluating the combinations of sample size versus number of steps, there is a shift from having no significant differences when using less than 20 steps to at least a 70% chance of finding significant results when using more than 20 runners and more than 20 steps. In addition, the number of runners highly influences the outcomes when using greater amounts of steps, and the number of steps is the major determinant of changes in effect size for the running speed.

A previous study comparing running patterns between treadmill and overground running using different shoe drops reported significant changes between running conditions, which were based on a different number of steps (20 treadmill steps and 7 overground steps)^34^. These authors reported a 51% increase in loading rate and greater foot contact angle during overground running when compared to treadmill running^34^. These authors stated that treadmill running is easier to accomplish whereas overground running presents greater intra-individual variability due to variable running speed (± 5%) and foot placement (within the force plate limits). Interestingly, other studies found no differences between treadmill and overground running variables analyzing a similar number of steps. Oliveira et al. (2016)^35^ have reported that the peak vertical tibial acceleration is similar between these conditions when using 20 running steps. However, these authors found greater variability in the vertical peak tibial acceleration during overground running. Moreover, Fellin et al. (2010)^36^ have reported no significant differences in lower limb kinematics (hip, knee, and ankle angle at foot strike and midstance) between the two conditions, reported from the same number of steps (5 running steps) in both conditions. Therefore, our results contribute to the current body of literature by establishing benchmarks for data variability and expected outcomes regarding sample size and number of steps for widely used running biomechanical variables.

Studies using a low number of steps to represent a runner’s performance may present the limitation of not achieving the required average stability. In this study, we found that a median of at least 15 steps is required to reach stability across all variables, for both normal and silent running (Fig. 4B). Moreover, the use of up to 10 steps dramatically reduced the number of runners reaching stable mean (< 35%), regardless of the biomechanical variable. This result is highly relevant for future studies, as the number of steps can influence the data representation of most of the runners, generating misleading results and interpretations. Our results demonstrated that a stable average for the majority of runners (> 50%) across all variables is achieved by using > 20 running steps, whereas each variable may require a different number of steps to reach stability (see Fig. 4C). A previous study comparing ground reaction force variables across different running shoes recorded 15–35 steps per runner^37^. However, only two steps per shoe were analyzed. The study reported between-gender differences, as well as increased injury risks depending on footwear^37^ for a sample size of (10 males and 10 females). Around 6 studies (11%) from our review have used less than 5 steps to describe running mechanics^19,28,38–41^. Based on our results, the use of fewer than 4 steps to report data from ground reaction force parameters might not provide ideal and/or stable intra-subject representation to describe the running biomechanical parameter. Therefore, the interpretation of results generated from low sample sizes and number of steps must be carried out with caution.

The reductions in variability for foot contact time, peak vertical force, and running speed (CV < 15%) in silent running may be related to substantial movement constrains in order to achieve reduced sound outputs. However, the vertical loading rate presented conflicting trends and increased variability during normal running. There was a greater coefficient of variation during normal running when evaluating more than 15 runners and steps, whereas for silent running there was a greater variability when evaluating a lower number of steps (< 25 steps) and runners (< 20 runners). These results are crucial to demonstrate that different running biomechanics variables may present unique trends within the same experimental design. Therefore, studies using several variables must account for the variability pattern of all of them when defining experimental procedures. It is noteworthy that variables expected to show differences between conditions (e.g., peak braking force and foot contact angle) presented substantial variability, especially during silent running (see Figs. 8 and 11, *central columns*). Again, the motor control demands to successfully achieve silent running may explain the larger variability for these variables^6^. Furthermore, runners were not instructed on how to achieve and/or perform silent running. It is suggested that each runner may have adopted a unique running style that was effective to reduce sound outputs, contributing to increases in inter-subject variability for some biomechanical variables. Therefore, studies investigating changes in running technique and/or motor control strategies should consider recording a sufficient number of steps in order to generate stable averages for appropriate representation of all described biomechanical variables. Furthermore, pilot studies involving several participants should be used to determine the preliminary minimum number of steps required to reach average stability across all targeted biomechanical variables. In this way, experimental designs will account for reaching stability across all variables.

This study has some limitations. Firstly, participants performed overground running in an indoor environment that may not be ideal for describing running kinematics/kinetics. The running track layout favored the repeated acquisition of running steps, but runners did not maintain constant speed over the course of the entire track. This was caused due to turns and changes in approaching speed to the force platform. In our study, the average running speed across all participants and conditions was approximately 2.7 m s^−1^. The vast majority of studies investigating novice runners report a running speed within 2.5 and 3.5 m s^−1^ (see P3, L79-94, and Fig. 1C), which contains the average running speed from this experiment. Since running biomechanical variables are speed-dependent, extrapolations of our results to running conditions where the running speed is outside this range should be done with caution. Secondly, another limitation of the study was the use of unfamiliar shoes by the runners. Despite the extensive procedures provided to runners to familiarize themselves with the shoes, the lack of long-term familiarity might affect the variability of the investigated biomechanical variables^38,42^. Future studies comparing the stability of biomechanical variables between short-term and long-term familiarization to running shoes are needed to address this limitation. Lastly, there was a large number of statistical comparisons in this study (> 600,000 paired tests) and another limitation of the study is the lack of correction for multiple comparisons. Due to the large scale of comparisons performed in this study, the possibility of type I errors cannot be overruled. Therefore, extrapolating the conclusions from our study to different types of shoes (e.g., motion-control shoes), running surfaces (outdoors or treadmill) and other populations (elite athletes) must be done with caution. Furthermore, the interpretation of the results should take into consideration that the task is performed indoors, therefore translating these results to outdoor conditions with changing terrains and/or environmental conditions might not be ideal.

In summary, our results suggest that the selection of both sample size and number of steps can influence the statistical outcomes for the comparison of normal *vs* silent running. Moreover, the influence of sample size and number of steps is variable-dependent. Therefore, our results provided experimental evidence to recommend that studies involving analysis of traditional running biomechanical variables use a minimum of 25 participants and 25 steps from each participant to increase the likelihood of describing accurate results with large effect sizes when comparing running in different conditions. These guidelines may assist researchers to design optimal experimental protocols in running biomechanics, as well as provide standard requirements to assess the quality of experimental outcomes in this research field.

## Supplementary Information

Supplementary Information 1.

Supplementary Information 2.
