# Supplementary material for: Implications of sample size and acquired number of steps to investigate running biomechanics
Source: Sci Rep. 2021 Feb 4;11:3083. doi: 10.1038/s41598-021-82876-z (PMC7862397; doi:10.1038/s41598-021-82876-z)
Supplement: Supplementary file 1 — Supplementary Information 1. [file 41598_2021_82876_MOESM1_ESM.docx]

**IMPLICATIONS OF SAMPLE SIZE AND ACQUIRED NUMBER OF STEPS TO INVESTIGATE RUNNING BIOMECHANICS**

Authors

Anderson Souza Oliveira

Cristina Ioana Pirscoveanu

**Supplementary table 1 – Experimental protocol and variability in running biomechanics studies**

A mini-literature review was conducted using PUBMED library of scientific studies published from January 2010 to October 2019 using the following terms “loading rate”, “running” and “ground reaction forces” liked with “and” operators. Additional filters were added after the initial search and articles with main research area involving the study of injuries, body weight unloading, literature reviews, walking, underaged participants and interventional devices were excluded from the review. Studies with similar methodology with our study and with the following parameters of interest were included: vertical average loading rate (VALR), vertical instant loading rate (VILR), foot contact angle at initial contact, contact time, peak braking force and running speed. A total of 14 articles were reviewed and their results extracted in order to obtain the coefficient of variation and effect size. Additional information regarding the sample size, analyzed trials as well as testing conditions were included in the review.

Table 1A. Characteristics of studies focused on running biomechanics.

| Study | Subjects | Steps | Speed m/s | Conditions |
| --- | --- | --- | --- | --- |
| Tate and Milner 2017 ^1^ | 14 | 5 | 2.96 ± 0.24 | Pre-training  ^a^ |
|  |  |  |  | Post-training ^b^ |
| Phan et al. 2017 ^2^ | 26 | 10 | 5.0 ± 0.5 | Normal ^a^ |
|  |  |  |  | Quiet ^b^ |
| Kluitenberg et al. 2012 ^3^ | 24 | 5 | 3.38 ± 0.45 | Heel stike ^a^ |
|  |  |  | 3.49 ± 0.45 | Non heel strike ^b^ |
| Tam et al. 2016 ^4^ | 51 | 6 | 3.5 ± 0.5 | Shod ^a^ |
|  |  |  |  | Barefoot ^b^ |
| Breine et al. 2017 ^5^ | 52 | 3 | 3.2 ± 0.2 | Reafoot strike ^a^ |
|  |  |  |  | Atypic reafoot strike ^b^ |
|  |  |  |  | Midfoot strike ^c^ |
| Willson et al. 2014 ^6^ | 19 | 5 | 3.7 ± 0.185 | Non-rearfoot strike ^a^ |
|  |  |  |  | Rearfoot strike ^b^ |
| Rice, Jamison and Davis 2016 ^7^ | 29 | 5 | 3.13 ± 0.15 | Forefoot strike ^a^ |
|  |  |  |  | Forefoot strike ^b^ |
|  |  |  |  | Midfoot stike ^c^ |
| Tam, Tucker, Wilson 2016 ^8^ | 29 | 6 | 3.5 ± 0.07 | Pre-training  ^a^ |
|  |  |  |  | Post-training ^b^ |
| Kuhman, Melcher, Paquette et al. 2016 ^9^ | 16 | 5 | 3.35 ± 0.16 ^1^ | Rearfoot strike ^a^ |
|  |  |  | 4.47 ± 0.22 ^2^ |  |
|  |  |  | 3.35 ± 0.16 ^1^ | Imposed rearfoot strike ^b^ |
|  |  |  | 4.47 ± 0.22 ^2^ |  |
| Logan et al. 2010 ^10^ | 20 | 15 to 35 | 5.7 ± 0.11 | Female ^a^ |
|  |  |  | 6.7 ± 0.13 | Male ^b^ |
| Mullineaux et al. 2006 ^11^ | 40 | 3 to 5 | 3.7 ± 0.18 | Female |
| Shih et al. 2019 ^12^ | 18 | 5 | 2.82 ± 0.06 | Pre-training ^a^ |
|  |  |  |  | Post-training ^b^ |
| Futrell et al. 2018 ^13^ | 32/125 | 10 | 2.58 ± 0.14 | Forefoot strike ^a^ |
|  |  |  | 2.59 ± 0.11 | Rearfoot strike ^b^ |
| Messier et al. 2018 ^14^ | 300 | 6 | 2.94 ± 22.35 | Injured ^a^ |
|  |  |  | 3.01 ± 22.35 | Uninjured ^b^ |

Table 1B. Analysis of included studies coefficient of variation (CV) and effect size (Cohen) for running speed, vertical average loading rate (VALR) and vertical instant loading rate (VILR).

| Study | Speed | | VALR | | VILR | |
| --- | --- | --- | --- | --- | --- | --- |
|  | **CV** | **Cohen** | **CV** | **Cohen** | **CV** | **Cohen** |
| Tate and Milner 2017 ^1^ | 8.10 |  | 29.1 ^a^ | 1.38 | 28.7 ^a^ | 1.31 |
|  |  |  | 36.7 ^b^ |  | 35.5 ^b^ |  |
| Phan et al. 2017 ^2^ | 10.00 |  | 54.8 ^a^ | 1.57 |  |  |
|  |  |  | 109.4 ^b^ |  |  |  |
| Kluitenberg et al. 2012 ^3^ | 12.78 ^a^ | 0.25 | 27.14 ^a^ | 2.12 |  |  |
|  | 12.93 ^b^ |  | 35.89 ^b^ |  |  |  |
| Tam et al. 2016 ^4^ | 14.28 |  |  |  | 58.93 ^a^ | 0.73 |
|  |  |  |  |  | 83.48 ^b^ |  |
| Breine et al. 2017 ^5^ | 6.25 |  |  |  |  |  |
| Willson et al. 2014 ^6^ | 4.99 |  | 21.04 ^a^ | 7.2 |  |  |
|  |  |  | 12.54 ^b^ |  |  |  |
| Rice, Jamison and Davis 2016 ^7^ | 4.79 |  |  |  | 38.94 ^a^ | 0.72 ^a,b^ |
|  |  |  |  |  | 25.74 ^b^ | 0.62 ^b,c^ |
|  |  |  |  |  | 25.47 ^c^ | 1.13 ^c,a^ |
| Tam, Tucker, Wilson 2016 ^8^ | 2.00 |  |  |  | 67.78 ^a^ | 0.27 |
|  |  |  |  |  | 39.52 ^b^ |  |
| Kuhman , Melcher, Paquette et al. 2016 ^9^ | 4.77 ^a1,b1^ | -5.82 ^a1,a2^ | 27.18 ^a1^ | -0.79 ^a1,a2^ |  |  |
|  |  |  | 26.50 ^a2^ |  |  |  |
|  | 4.921 ^a2,b2^ | -5.82 ^b1,b2^ | 22.39 ^b1^ | -0.94 ^b1,b2^ |  |  |
|  |  | 0 ^a,b^ | 29.90 ^b2^ | 2.18 ^a,b^ |  |  |
| Logan et al. 2010 ^10^ | 1.93 ^a^ | 8.30 | 36.49 ^a^ | -0.06 |  |  |
|  | 1.94 ^b^ |  | 31.13 ^b^ |  |  |  |
| Mullineaux et al. 2006 ^11^ | 4.86 |  | 31.62 |  | 26.94 |  |
| Shih et al. 2019 ^12^ | 2.12 |  | 35.04 ^a^ | 0.04 |  |  |
|  |  |  | 38.31 ^b^ |  |  |  |
| Futrell et al. 2018 ^13^ | 5.42 ^a^ | -0.07 | 29.54 |  | 25.64 |  |
|  | 4.24 ^b^ |  |  |  |  |  |
| Messier et al. 2018 ^14^ | 760.2 ^a^ | -0.003 |  |  |  |  |
|  | 742.52 ^b^ |  |  |  |  |  |

Table 1C. Analysis of included studies coefficient of variation (CV) and effect size (Cohen) for foot contact angle at initial contact, contact time and peak braking force.

| Study | Foot angle at initial contact | | Contact time | | Peak braking force | |
| --- | --- | --- | --- | --- | --- | --- |
|  | **CV** | **Cohen** | **CV** | **Cohen** | **CV** | **Cohen** |
| Tate and Milner 2017 ^1^ |  |  |  |  |  |  |
| Phan et al. 2017 ^2^ | 3388.23 ^a^ | 1.15 | 10 ^a^ | 0 |  |  |
|  | 106.41 ^b^ |  | 10 ^b^ |  |  |  |
| Kluitenberg et al. 2012 ^3^ |  |  | 9.33 ^a^ | 0.56 |  |  |
|  |  |  | 6.73 |  |  |  |
| Tam et al. 2016 ^4^ | 111.63 ^a^ | 1.1 |  |  |  |  |
|  | -480 ^b^ |  |  |  |  |  |
| Breine et al. 2016 ^5^ | 23.52 ^a^ | 2.71 ^a,b^ | 6.22 ^a^ | 0.88 ^a,b^ |  |  |
|  | 72.85 ^b^ | 1.28 ^b,c^ | 6.66 ^b^ | 0.13 ^b,c^ |  |  |
|  | 193.75 ^c^ | 4.65 ^c,a^ | 6.72 ^c^ | 1 ^c,a^ |  |  |
| Wilson et al. 2014 ^6^ | 34.05 ^a^ | 6.19 |  |  |  |  |
|  | 28.3 ^b^ |  |  |  |  |  |
| Rice, Jamison and Davis 2016 ^7^ |  |  | 8.52 ^a^ | 1.11 (a,b) |  |  |
|  |  |  | 8.13 | -0.89 (b,c) |  |  |
|  |  |  | 3.85 | 0.56 (c,a) |  |  |
| Tam, Tucker, Wilson 2016 ^8^ | 95.95 ^a^ | 0.06 | 7.41 ^a^ | -0.5 |  |  |
|  | 93.67 ^b^ |  | 7.14 ^b^ |  |  |  |
| Kuhman , Melcher, Paquette et al. 2016 ^9^ |  |  |  |  |  |  |
|  |  |  |  |  |  |  |
| Logan et al. 2010 ^10^ |  |  | 5.39 ^a^ | -0.89 | -28.57 ^a^ | 0.17 |
|  |  |  | 8.28 ^b^ |  | -20.90 ^b^ |  |
| Mullineaux et al. 2006 ^11^ |  |  |  |  |  |  |
| Shih et al. 2019 ^12^ | -169.59 ^a^ | -0.82 |  |  |  |  |
|  | 371.93 ^b^ |  |  |  |  |  |
| Futrell et al. 2018 ^13^ |  |  |  |  |  |  |
| Messier et al. 2018 ^14^ | 61.66 ^a^ | -0.1 |  |  | 25 ^a^ | -0.21 |
|  | 56.25 ^b^ |  |  |  | 23.25 ^b^ |  |

**References**

1. Tate, J. J. & Milner, C. E. Sound-Intensity Feedback During Running Reduces Loading Rates and Impact Peak. *J. Orthop. Sports Phys. Ther.* **47**, 565–569 (2017).

2. Phan, X. *et al.* Running quietly reduces ground reaction force and vertical loading rate and alters foot strike technique. *J. Sports Sci.* **35**, 1636–1642 (2017).

3. Kluitenberg, B., Bredeweg, W, S., Zijlstra, S., Zijlstra, W. & Buist, I. Comparison of vertical ground reaction forces during overground and treadmill running. A validation study. *BMC Musculoskelet. Disord.* **13**, 235 (2012).

4. Tam, N., Astephen Wilson, J. L., Coetzee, D. R., van Pletsen, L. & Tucker, R. Loading rate increases during barefoot running in habitually shod runners: Individual responses to an unfamiliar condition. *Gait Posture* **46**, 47–52 (2016).

5. Breine, B. *et al.* Initial foot contact and related kinematics affect impact loading rate in running. *J. Sports Sci.* **35**, 1556–1564 (2017).

6. Willson, J. D. *et al.* Short-Term Changes in Running Mechanics and Foot Strike Pattern After Introduction to Minimalistic Footwear. *PM R* **6**, 34–43 (2014).

7. Rice, D., Jamison, S. & Davis, I. Footwear Matters: Influence of Footwear and Foot Strike on Load Rates during Running. *Med. Sci. Sport. Exerc.* **48**, 2462–2468 (2016).

8. Tam, N., Tucker, R. & Astephen Wilson, J. L. Individual Responses to a Barefoot Running Program. *Am. J. Sports Med.* **44**, 777–784 (2016).

9. Kuhman, D., Melcher, D. & Paquette, M. R. Ankle and knee kinetics between strike patterns at common training speeds in competitive male runners. *Eur. J. Sport Sci.* **16**, 433–440 (2016).

10. Logan, S., Hunter, I., Hopkins, J. T., Feland, J. B. & Parcell, A. C. Ground reaction force differences between running shoes, racing flats, and distance spikes in runners. *J. Sport. Sci. Med.* **9**, 147–153 (2010).

11. Mullineaux, D., Milner, C., Davis, I. & Hamill, J. Normalization of Ground Reaction Forces. *J. Appl. Biomech.* **22**, 230–233 (2006).

12. Shih, H. T. *et al.* Four weeks of training with simple postural instructions changes trunk posture and foot strike pattern in recreational runners. *Phys. Ther. Sport* **35**, 89–96 (2019).

13. Futrell, E. E., Jamison, S. T., Tenforde, A. S. & Davis, I. S. Relationships between Habitual Cadence, Footstrike, and Vertical Load Rates in Runners. *Med. Sci. Sports Exerc.* **50**, 1837–1841 (2018).

14. Messier, S. P. *et al.* A 2-Year Prospective Cohort Study of Overuse Running Injuries: The Runners and Injury Longitudinal Study (TRAILS). *Am. J. Sports Med.* **46**, 2211–2221 (2018).
