# Supplementary material for: Implications of sample size and acquired number of steps to investigate running biomechanics
Source: Sci Rep. 2021 Feb 4;11:3083. doi: 10.1038/s41598-021-82876-z (PMC7862397; doi:10.1038/s41598-021-82876-z)
Supplement: Supplementary file 2 — Supplementary Information 2. [file 41598_2021_82876_MOESM2_ESM.docx]

**IMPLICATIONS OF SAMPLE SIZE AND ACQUIRED NUMBER OF STEPS TO INVESTIGATE RUNNING BIOMECHANICS**

Authors

Anderson Souza Oliveira

Cristina Ioana Pirscoveanu

**Supplementary table 2 – Review of running biomechanics studies from 2000 until present.**

A review was conducted using PUBMED library of scientific studies published from 2010 to November 2019 using the following terms “loading rate”, “running” and “ground reaction forces” liked with “and” operators. Additional filters were added after the initial search and articles with main research area involving the study of injuries, body weight unloading, literature reviews, walking, underaged participants and interventional devices were excluded from the review. A total of 56 articles were reviewed to determine their methodology and extract the number of participants, trials or steps and running speed used to quantify their results. Additional information regarding the studies’ testing conditions and footwear type were included.

Table 2. Characteristics of studies focused on running biomechanics from 2000 until present.

| Studies | Subjects | Steps | Speed (m/s) | Running conditions | Running  footwear |
| --- | --- | --- | --- | --- | --- |
| Christina et al. 2001 ^1^ | 11 | 5 | 2.9 | treadmill | shod |
| Mullineaux et al. 2006 ^2^ | 40 | 3-5 | 3.7 | overground | - |
| O'Leary et al. 2008 ^3^ | 16 | 5 | 3.2 ± 0.3 | overground | shod |
| Logan et al. 2010 ^4^ | 20 | 15-35 | 5.7 (females)  6.7 (males) | track | shod |
| Lilley et al. 2011 ^5^ | 30 | 10 | 3.5 | overground | shod |
| Morin et al. 2011 ^6^ | 10 | 10 | 2.77 | tredmill | - |
| Kluitenberg et al.2012 ^7^ | 24 | 5 | 3.05 ± 0.36;  3.0 ± 0.41:  3.52 ± 0.44;  3.27 ± 0.41;  3.91 ± 0.55;  3.86 ± 0.52; | both | shod |
| Hobara et al. 2012 ^8^ | 10 | 10 | 2.5 | tredmill | - |
| Rowlands and Stiles 2012 ^9^ | 10 | 8 | self-selected | overground | - |
| Degache et al. 2012 ^10^ | 8 | 10 | 2.77;  3.33;  3.88; | treadmill | - |
| Chumanov et al .2012 ^11^ | 45 | 5 | 2.9 ± 0.5 | treadmill | - |
| Gross & Gross 2013 ^12^ | 22 | 5 | 2.87 ± 0.45;  2.53 ± 0.48; | treadmill | shod |
| Stiles et al. 2013 ^13^ | 47 | 8 | 130-140 steps/min;  145-160 steps/min; | overground | - |
| Stiles et al. 2013 ^14^ | 8 | 10 | 3.83 | overground | shod |
| Bredeweg et al.2013 ^15^ | 210 | 10 | 2.22;  2.5;  2.77; | treadmill | - |
| Breine et al. 2013 ^16^ | 55 | 3 | 3.2;  4.1;  5.1;  6.2; | overground | both |
| Willy and Davis 2013 ^17^ | 14 | 5 | 3.35 | treadmill | shod |
| Wille et al. 2014 ^18^ | 45 | 5 | 2.94± 0.42 | treadmill | - |
| Willson et al. 2014 ^19^ | 19 | 5 | 3.52;  3.89; | overground | shod |
| Chambon et al. 2014 ^20^ | 15 | 5 | 3.3 | overground | both |
| Lenhart et al. 2014 ^21^ | 30 | 5 | 2.4-3.8 | treadmill | shod |
| Chambon et al. 2015 ^22^ | 20 | 20 and 7 | > 2.22 | both | shod |
| Kline and Williams 2015^23^ | 41 | 10 | 3.3 ± 0.2 | overground | shod |
| Willson et al. 2015 ^24^ | 35 | 5 | 3.5 | treadmill | shod |
| Goss et al. 2015 ^25^ | 60 | 5 | 2.7-2.9 | treadmill | - |
| Kobayashi et al. 2016 ^26^ | 9 | 10 | 2.5;  3;  3.5; | treadmill | shod |
| Tam et al. 2016^27^ | 51 | 6 | 3.5 ± 0.5 | track | Both |
| Studies | **Subjects** | **Steps** | **Speed (m/s)** | **Running conditions** | **Running**  **footwear** |
| Breine et al .2016 ^28^ | 52 | 3 | 3.2 | overground | shod |
| Rice et al. 2016 ^29^ | 29 | 5 | 3.13 ± 0.15 | overground | shod |
| Tam et al. 2016 ^30^ | 29 | 6 | 3.5 ± 0.07 | overground | both |
| Kuhman et al. 2016 ^31^ | 16 | 5 | 3.5 | overground | - |
| Kuhman et al .2016 ^32^ | 24 | 10 | 4.5 ± 0.23(men);  4.0 ± 0.2 (women); | track | shod |
| Fu et al. 2016 ^33^ | 11 | 5 | 2.50 ± 0.14;  3.05 ± 0.1;  2.24 ± 0.26;  2.84 ± 0.29; | overground | shod |
| Jewell et al. 2017 ^34^ | 14 | 5 | 3.88 ± 0.60 | overground | - |
| Phan et al. 2017 ^35^ | 26 | 10 | 5.0 ± 0.5 | overground | barefoot |
| Tate and Milner 2017 ^36^ | 14 | 5 | 2.96 ± 0.24 | both | - |
| Tam et al. 2017 ^37^ | 22 | 6 | 3.5;  4.4; | overground | both |
| Firminger et al. 2018 ^38^ | 11 | 5 | treadmill  2.13±0.22;  2.61±0.14;  3.27±0.22;  overground  2.61±0.14;  3.44±0.14;  4.22±0.14 | both | shod |
| Arnold et al. 2018 ^39^ | 26 | 5 | 2.51 ± 0.30 (barefoot);  2.48 ± 0.14 (shod); | treadmill | shod |
| Kulmala et al. 2018 ^40^ | 12 | 5 | 2.77 ± 0.08 ;  4.02 ± 0.08 ; | overground | shod |
| Bazuelo-Ruiz et al. 2018 ^41^ | 57 | 5 | 3.3 | overground | shod |
| Wouda et al. 2018 ^42^ | 18 | 200 | 2.77;  3.33;  3.88; | treadmill | shod |
| Pollard et al. 2018 ^43^ | 15 | 5 | self-selected | overground | shod |
| Breine et al. 2018 ^44^ | 52 | 3 | 3.2;  4.1;  5.1;  6.2; | overground | shod |
| Renner et al. 2019 ^45^ | 30 | 36.2  35.7 | 3.0;  3.5; | treadmill insoles | shod |
| Matijevich et al. 2019 ^46^ | 10 | 30 | 2.6;  2.8;  3.0;  3.2;  3.4;  3.6;  3.8;  4.0 ; | treadmill | shod |
| Studies | **Subjects** | **Steps** | **Speed (m/s)** | **Running conditions** | **Running footwear** |
| Stiffler-Joachim et al. 2019 ^47^ | 170 | 15 | 2.68;  3.35;  4.47; | treadmill | shod |
| Shih et al. 2019 ^48^ | 40 | 5 | 3.4 | overground | shod |
| Verheul et al. 2019 ^49^ | 15 | 5 | 2-3;  4-5;  >6 ; | overground | shod |
| Zhang et al. 2019 ^50^ | 20 | 10 | 3.5 | treadmill | shod |
| White et al. 2019 ^51^ | 10 | 5 | 3.01 ± 0.4 | treadmill | shod |
| Hanningan and Pollard 2019 ^52^ | 20 | 5 | 2.85 ± .07 | overground | shod |
| Jafarnezhadgero et al. 2019 ^53^ | 22 | 5 | 3.3 | overground | shod |
| Hanningan and Pollard 2019 ^54^ | 28 | 5 | 3.35 | overground | shod |
| Van der Berghe et al. 2019 ^55^ | 13 | 4 | 2.55 ± 0.2;  3.20 ± 0.2;  5.10 ± 0.2; | overground | shod |
| Khassetarash et al. 2019 ^56^  References | 11 | 4 | 4.29 ± 0.33 | both | - |

1. Christina, K. A., White, S. C. & Gilchrist, L. A. Effect of localized muscle fatigue on vertical ground reaction forces and ankle joint motion during running. *Hum. Mov. Sci.* **20**, 257–276 (2001).

2. Mullineaux, D., Milner, C., Davis, I. & Hamill, J. Normalization of Ground Reaction Forces. *J. Appl. Biomech.* **22**, 230–233 (2006).

3. O’Leary, K., Vorpahl, K. A. & Heiderscheit, B. Effect of cushioned insoles on impact forces during running. *J. Am. Podiatr. Med. Assoc.* **98**, 36–41 (2008).

4. Logan, S., Hunter, I., Hopkins, J. T., Feland, J. B. & Parcell, A. C. Ground reaction force differences between running shoes, racing flats, and distance spikes in runners. *J. Sport. Sci. Med.* **9**, 147–153 (2010).

5. Lilley, K., Dixon, S. & Stiles, V. A biomechanical comparison of the running gait of mature and young females. *Gait Posture* **33**, 496–500 (2011).

6. Morin, J. B., Samozino, P. & Millet, G. Y. Changes in running kinematics, kinetics, and spring-mass behavior over a 24-h run. *Med. Sci. Sports Exerc.* **43**, 829–836 (2011).

7. Kluitenberg, B., Bredeweg, W, S., Zijlstra, S., Zijlstra, W. & Buist, I. Comparison of vertical ground reaction forces during overground and treadmill running. A validation study. *BMC Musculoskelet. Disord.* **13**, 235 (2012).

8. Hobara, H., Sato, T., Sakaguchi, M., Sato, T. & Nakazawa, K. Step frequency and lower extremity loading during running. *Int. J. Sports Med.* **33**, 310–313 (2012).

9. Rowlands, A. V. & Stiles, V. H. Accelerometer counts and raw acceleration output in relation to mechanical loading. *J. Biomech.* **45**, 448–454 (2012).

10. Degache, F. *et al.* Changes in running mechanics and spring-mass behaviour induced by a 5-hour hilly running bout. *J. Sports Sci.* **31**, 299–304 (2012).

11. Chumanov, E. S., Wille, C. M., Michalski, M. P. & Heiderscheit, B. C. Changes in muscle activation patterns when running step rate is increased. *Gait Posture* **36**, 231–235 (2012).

12. Goss, D. L. & Gross, M. T. A comparison of negative joint work and vertical ground reaction force loading rates in chi runners and rearfoot-striking runners. *J. Orthop. Sports Phys. Ther.* **43**, 685–692 (2013).

13. Stiles, V. H., Griew, P. J. & Rowlands, A. V. Use of accelerometry to classify activity beneficial to bone in premenopausal women. *Med. Sci. Sports Exerc.* **45**, 2353–2361 (2013).

14. Stiles, V. H., Guisasola, I. N., James, I. T. & Dixon, S. J. Biomechanical response to changes in natural turf during running and turning. *J. Appl. Biomech.* **27**, 54–63 (2013).

15. Bredeweg, S. W., Kluitenberg, B., Bessem, B. & Buist, I. Differences in kinetic variables between injured and noninjured novice runners: A prospective cohort study. *J. Sci. Med. Sport* **16**, 205–210 (2013).

16. Breine, B., Malcolm, P., Frederick, E. C. & De Clercq, D. Relationship between running speed and initial foot contact patterns. *Med. Sci. Sports Exerc.* **46**, 1595–1603 (2013).

17. Willy, R. W. & Davis, I. S. Kinematic and kinetic comparison of running in standard and minimalist shoes. **46**, 318–323 (2013).

18. Wille, C. M., Lenhart, R. L., Wang, S., Thelen, D. G. & Heiderscheit, B. C. Ability of sagittal kinematic variables to estimate ground reaction forces and joint kinetics in running. *J. Orthop. Sports Phys. Ther.* **44**, 825–830 (2014).

19. Willson, J. D. *et al.* Short-Term Changes in Running Mechanics and Foot Strike Pattern After Introduction to Minimalistic Footwear. *PM R* **6**, 34–43 (2014).

20. Chambon, N., Delattre, N., Guéguen, N., Berton, E. & Rao, G. Is midsole thickness a key parameter for the running pattern? *Gait Posture* **40**, 58–63 (2014).

21. Lenhart, R., Thelen, D. & Heiderscheit, B. Hip muscle loads during running at various step rates. *J. Orthop. Sports Phys. Ther.* **44**, 766–774 (2014).

22. Chambon, N., Delattre, N., Guéguen, N., Berton, E. & Rao, G. Shoe drop has opposite influence on running pattern when running overground or on a treadmill. *Eur. J. Appl. Physiol.* **115**, 911–918 (2015).

23. Kline, P. W. & Williams, D. S. B. Effects of Normal Aging on Lower Extremity Loading and Coordination During Running in Males and Females. *Int. J. Sports Phys. Ther.* **10**, 901–9 (2015).

24. Willson, J. D., Loss, J. R., Willy, R. W. & Meardon, S. A. Sex differences in running mechanics and patellofemoral joint kinetics following an exhaustive run. *J. Biomech.* **48**, 4155–4159 (2015).

25. Goss, D. L. *et al.* Lower extremity biomechanics and self-reported foot-strike patterns among runners in traditional and minimalist shoes. *J. Athl. Train.* **50**, 603–611 (2015).

26. Kobayashi, Y. *et al.* Comparison of 3 Methods for Computing Loading Rate during Running. *Int. J. Sports Med.* **37**, 1087–1090 (2016).

27. Tam, N., Astephen Wilson, J. L., Coetzee, D. R., van Pletsen, L. & Tucker, R. Loading rate increases during barefoot running in habitually shod runners: Individual responses to an unfamiliar condition. *Gait Posture* **46**, 47–52 (2016).

28. Breine, B. *et al.* Initial foot contact and related kinematics affect impact loading rate in running. *J. Sports Sci.* **35**, 1556–1564 (2017).

29. Rice, D., Jamison, S. & Davis, I. Footwear Matters: Influence of Footwear and Foot Strike on Load Rates during Running. *Med. Sci. Sport. Exerc.* **48**, 2462–2468 (2016).

30. Tam, N., Tucker, R. & Astephen Wilson, J. L. Individual Responses to a Barefoot Running Program. *Am. J. Sports Med.* **44**, 777–784 (2016).

31. Kuhman, D., Melcher, D. & Paquette, M. R. Ankle and knee kinetics between strike patterns at common training speeds in competitive male runners. *Eur. J. Sport Sci.* **16**, 433–440 (2016).

32. Kuhman, D. J., Paquette, M. R., Peel, S. A. & Melcher, D. A. Comparison of ankle kinematics and ground reaction forces between prospectively injured and uninjured collegiate cross country runners. *Hum. Mov. Sci.* **47**, 9–15 (2016).

33. Fu, F. *et al.* Lower limb mechanics during moderate high-heel jogging and running in different experienced wearers. *Hum. Mov. Sci.* **48**, 15–27 (2016).

34. Jewell, C., Boyer, K. A. & Hamill, J. Do footfall patterns in forefoot runners change over an exhaustive run? *J. Sports Sci.* **35**, 74–80 (2017).

35. Phan, X. *et al.* Running quietly reduces ground reaction force and vertical loading rate and alters foot strike technique. *J. Sports Sci.* **35**, 1636–1642 (2017).

36. Tate, J. J. & Milner, C. E. Sound-Intensity Feedback During Running Reduces Loading Rates and Impact Peak. *J. Orthop. Sports Phys. Ther.* **47**, 565–569 (2017).

37. Tam, N. *et al.* Acute fatigue negatively affects risk factors for injury in trained but not well-trained habitually shod runners when running barefoot. *Eur. J. Sport Sci.* **17**, 1220–1229 (2017).

38. Firminger, C. R. *et al.* Joint kinematics and ground reaction forces in overground versus treadmill graded running. *Gait Posture* **63**, 109–113 (2018).

39. Arnold, B. J. W., Weeks, B. K. & Horan, S. A. An examination of treadmill running familiarisation in barefoot and shod conditions in healthy men. *J. Sports Sci.* **37**, 5–12 (2018).

40. Kulmala, J. P., Kosonen, J., Nurminen, J. & Avela, J. Running in highly cushioned shoes increases leg stiffness and amplifies impact loading. *Sci. Rep.* **8**, 1–7 (2018).

41. Bazuelo-Ruiz, B., Durá-Gil, J. V., Palomares, N., Medina, E. & Llana-Belloch, S. Effect of fatigue and gender on kinematics and ground reaction forces variables in recreational runners. *PeerJ* **2018**, 1–12 (2018).

42. Wouda, F. J. *et al.* Estimation of vertical ground reaction forces and sagittal knee kinematics during running using three inertial sensors. *Front. Physiol.* **9**, 1–14 (2018).

43. Pollard, C. D., Ter Har, J. A., Hannigan, J. J. & Norcross, M. F. Influence of Maximal Running Shoes on Biomechanics Before and After a 5K Run. *Orthop. J. Sport. Med.* **6**, 1–5 (2018).

44. Breine, B. *et al.* Running speed-induced changes in foot contact pattern influence impact loading rate. *Eur. J. Sport Sci.* **19**, 774–783 (2019).

45. Renner, K. E., Blaise Williams, D. S. & Queen, R. M. The reliability and validity of the Loadsol® under various walking and running conditions. *Sensors (Switzerland)* **19**, 1–14 (2019).

46. Matijevich, E. S., Branscombe, L. M., Scott, L. R. & Zelik, K. E. Ground reaction force metrics are not strongly correlated with tibial bone load when running across speeds and slopes: Implications for science, sport and wearable tech. *PLoS One* **14**, 1–19 (2019).

47. Stiffler-Joachim, M. R., Wille, C. M., Kliethermes, S. A., Johnston, W. & Heiderscheit, B. C. Foot Angle and Loading Rate during Running Demonstrate a Nonlinear Relationship. *Med. Sci. Sports Exerc.* **51**, 2067–2072 (2019).

48. Shih, H. T. *et al.* Four weeks of training with simple postural instructions changes trunk posture and foot strike pattern in recreational runners. *Phys. Ther. Sport* **35**, 89–96 (2019).

49. Verheul, J., Gregson, W., Lisboa, P., Vanrenterghem, J. & Robinson, M. A. Whole-body biomechanical load in running-based sports: The validity of estimating ground reaction forces from segmental accelerations. *J. Sci. Med. Sport* **22**, 716–722 (2019).

50. Zhang, J. H., Chan, Z. Y. S., Au, I. P. H., An, W. W. & Cheung, R. T. H. Gait & Posture Can runners maintain a newly learned gait pattern outside a laboratory environment following gait retraining ? *Gait Posture* **69**, 8–12 (2019).

51. White, J. D., Carson, N., Baum, B. S., Reinking, M. F. & McPoil, T. G. Use of 2-Dimensional Sagittal Kinematic Variables To Estimate Ground Reaction Force During Running. *Int. J. Sports Phys. Ther.* **14**, 174–179 (2019).

52. Hannigan, J. J. & Pollard, C. D. Differences in running biomechanics between a maximal, traditional, and minimal running shoe. *J. Sci. Med. Sport* **23**, 15–19 (2019).

53. Jafarnezhadgero, A. A., Sorkhe, E. & Oliveira, A. S. Motion-control shoes help maintaining low loading rate levels during fatiguing running in pronated female runners. *Gait Posture* **73**, 65–70 (2019).

54. Hannigan, J. J. & Pollard, C. D. A 6-Week Transition to Maximal Running Shoes Does Not Change Running Biomechanics. *Am. J. Sports Med.* **47**, 968–973 (2019).

55. Van den Berghe, P., Six, J., Gerlo, J., Leman, M. & De Clercq, D. Validity and reliability of peak tibial accelerations as real-time measure of impact loading during over-ground rearfoot running at different speeds. *J. Biomech.* **86**, 238–242 (2019).

56. Khassetarash, A., Hassannejad, R., Ettefagh, M. M. & Oskouei, A. E. Vibration settling time of the gastrocnemius remains constant during an exhaustive run in rear foot strike runners. *J. Biomech.* **93**, 140–146 (2019).
